# Coherent Phase Change in Interstitial Solutions: A Hierarchy of Instabilities

**DOI:** 10.1002/advs.202308554

**Published:** 2024-03-21

**Authors:** Jörg Weissmüller

**Affiliations:** ^1^ Institute of Materials Physics and Technology Hamburg University of Technology 20173 Hamburg Germany; ^2^ Institute of Materials Mechanics Helmholtz‐Zentrum Hereon 21502 Geesthacht Germany

**Keywords:** Bitter‐Crum theorem, coherent phase transformations, metal hydrides, open‐system elasticity, phase transformation mechanisms, battery electrodes

## Abstract

Metal hydrides or lithium ion battery electrodes can take the form of interstitial solid solutions with a miscibility gap. This work discusses theory approaches for locating, in temperature‐composition space, coherent phase transformations during the charging/discharging of such systems and for identifying the associated transformation mechanisms. The focus is on the simplest scenario, where instabilities derive from the thermodynamics of the bulk phase alone, considering strain energy as the foremost consequence of coherency and admitting for stress relaxation at free surfaces. The extension of the approach to include capillarity is demonstrated by an example. The analysis rests on constrained equilibrium phase diagrams that are informed by geometry‐ and dimensionality‐specific mechanical boundary conditions and on elastic instabilities–again geometry‐specific–as implied by the theory of open‐system elasticity. It is demonstrated that some scenarios afford the analysis of chemical stability to be based entirely on a linear stability analysis of the mechanical equilibrium, which provides closed‐form solutions in a straightforward manner. Attention is on the impact of the system geometry (infinitely extended or of finite size) and on the chemical (closed or open system) and mechanical (incoherent or coherent) boundary conditions. Transformation mechanism maps are suggested for documenting the findings. The maps reveal a hierarchy of instabilities, which depend strongly on each of the above characteristics. Specifically, realistic, finite‐sized systems differ qualitatively from idealized systems of infinite extension. Among the transformation mechanisms exposed by the analysis are a uniform switchover to the other phase when the open system reaches its chemical spinodal, practical coherent nucleation, as well as chemo‐elastically coupled spontaneous buckling modes, which may take the form of either, single‐phase or dual‐phase states.

## Graphical abstract

Transformation mechanism maps document instabilities that trigger coherent phase transformations in battery electrodes or metal hydrides. The instabilities can arise from sign changes of specific open‐system elastic parameters, at readily determined temperatures and compositions. Analyzing the onset criteria for chemical disproportionation in alloys from a mechanics—and not chemistry—perspective challenges a paradigm in the theory of coherent transformations.

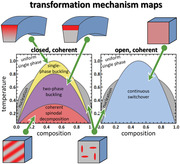

## 1 Introduction

The reversible insertion and removal of interstitials in crystalline host substrates has wide applications in modern energy storage schemes. Interstitials distort the host lattice, and the associated, parabolic composition‐dependence of the misfit strain energy^[^ ^1^, ^2^ ^]^ favors miscibility gaps.^[^ ^3^ ^]^ Interstitial solid solutions with a miscibility gap are exemplified by metal hydrides^[^ ^4^, ^5^ ^]^ and by lithium‐ion battery electrodes materials such as ${\mathrm{LiFePO}}_{4}$.^[^ ^6^, ^7^, ^8^ ^]^ Scenarios in which the crystalline coherency is maintained when cycling through the gap can be particularly favorable, as they avoid the accumulation of lattice defects and the ensuing degradation. Equilibrium conditions and transformation kinetics are then strongly affected by coherency strain energy. In a classic approach, transformation pathways and equilibria are deduced based on free energy minimization, starting with the stress‐free free energy functions of the coexisting phases and adding a coherency strain energy function as a correction term.^[^ ^9^, ^10^, ^11^, ^12^, ^13^, ^14^ ^]^ Yet, tangible expressions for the location of coherent phase transformations in temperature‐composition space can be elusive, and many studies resort to numerical approaches.^[^ ^15^, ^16^, ^17^, ^18^, ^19^, ^20^, ^21^, ^22^, ^23^, ^24^ ^]^

The Bitter‐Crum theorem^[^ ^25^, ^26^, ^27^ ^]^—according to which solute atoms in an infinitely extended and isotropic closed system do not interact—may simplify the analysis and has enabled important predictions.^[^ ^25^, ^28^ ^]^ Yet, the theorem requires infinitely extended systems,^[^ ^15^, ^26^ ^]^ excluding relaxation at free surfaces. This is a concern for real‐world and specifically for nanoscale systems. Spontaneous buckling during alloying, reported for certain nanomaterials,^[^ ^18^, ^29^ ^]^ has no equivalent in infinitely extended systems. The distinction between coherent phase transformations in Bitter‐Crum‐type and in finite‐size systems has not been explored in detail.

As for transformation pathways, instability against the spontaneous formation and growth of the new phase has been suggested,^[^ ^21^, ^28^ ^]^ yet the relevance of nucleation and growth^[^ ^30^, ^31^ ^]^ along with interfacial mobility^[^ ^32^ ^]^ were also emphasized. Specifically for the rapid lithiation of ${\mathrm{FePO}}_{4}$ particles, experiment and theory agree on a solid‐state wetting transition initiating the transformation.^[^ ^33^, ^34^, ^35^ ^]^ As different mechanisms of transformation can act, depending on the material and on boundary conditions such as system size, temperature, composition and driving force, there is an interest in exploring general principles by which the coupling between chemistry and mechanics acts to select the transformation mechanism, depending on the boundary condition, and in mapping the results for illustrative model scenarios.

In battery electrode materials, transformation paths that maintain coherency while suppressing phase coexistence are observed for rapid lithiation.^[^ ^17^, ^24^, ^35^, ^36^, ^37^, ^38^, ^39^, ^40^, ^41^ ^]^ The crucial role of the ratio (quantified by the “Damköhler number”, *Da*) of two time constants for composition change has been emphasized, one relating to the injection or removal of solute through the interface, and the other relating to the internal solute redistribution by bulk diffusion.^[^ ^40^ ^]^ High values of *Da* and a dependency of the exchange current density during rapid lithiation on the surface composition can be linked to the suppression of phase coexistence.^[^ ^40^, ^41^, ^42^ ^]^

The morphology of coherent phase patterns can be affected by external traction forces. Service loads may induce rafting in superalloy turbine blades,^[^ ^26^, ^43^ ^]^ epitaxy stresses may promote spinodal decomposition in thin semiconductor films^[^ ^44^ ^]^ and interparticle interactions may control domain patterns in powder‐based cathode materials.^[^ ^45^ ^]^ Yet, the internal coherency stresses in simple scenarios already provide a rich phenomenology.

Empirical findings link coherent transformations to small size.^[^ ^32^, ^46^, ^47^ ^]^ The large area‐per‐volume of small systems and their short diffusion pathways permit rapid changes in composition that may allow incoherent nucleation events to be bypassed. Furthermore, small systems may be statistically free of lattice defects^[^ ^48^ ^]^ that would serve as heterogeneous nucleation sites. Lastly, the enhanced yield strength at small size impedes the nucleation–required for incoherent precipitation–of lattice dislocations.^[^ ^46^, ^49^ ^]^ Thus, high driving forces, sufficient for coherent transformation, may be maintained at small size. By contrast, the magnitude of the required driving force is not forcefully and inherently size‐dependent. This is exemplified by the ultimate instability of an open system when the chemical potential reaches the bulk spinodal,^[^ ^50^ ^]^ which underlies continuous transitions without phase coexistence that retain the coherency;^[^ ^35^, ^36^, ^37^, ^39^, ^40^ ^]^ this transformation pathway is even compatible with periodic boundary conditions that emulate infinitely extended systems.^[^ ^22^ ^]^ A further example is the Schwarz–Khachaturyan instability against the discontinuous formation of a coherent new phase, which relies on a Bitter‐Crum‐type, size‐independent analysis of the coherency strain energy.^[^ ^28^ ^]^ Yet, size‐dependent driving forces or compositions for coherent phase change have also been repeatedly demonstrated.^[^ ^17^, ^51^, ^52^, ^53^, ^54^, ^55^ ^]^

The present work aims at a predictive theory for the location, in temperature‐composition space, of coherent phase transformation onsets and for the nature of the associated transformation mechanisms. Once the transformation is initiated, the kinetics by which it propagates are not the subject of this study. For conciseness, the focus is on the foremost consequence of coherency, namely the extra energy due to the misfit strain in the bulk. While free surfaces in finite‐sized systems provide for mechanical relaxation, the equations of bulk continuum mechanics are scale‐independent. Size effects may arise from capillarity–distinct materials behavior and free energy functions at surfaces or internal interfaces–and this is admitted in one exemplary scenario.

The analysis is based on two approaches, namely identifying i) equilibria with coherently coexisting states that may require thermally activated nucleation, and ii) instabilities towards phase change, as revealed by a linear stability analysis of the mechanics in systems with mobile solute and, hence, open‐system elasticity. Transformation mechanism maps are advertised as a convenient means of communicating the insights.

## 2 Fundamentals

### 2.1 Free Energy Functions

We consider an elastic interstitial solid solution with constant amounts of the host component and, therefore, constant amount *N*_0_ of sites for interstitials. The underlying concept is that of the network solid of Larché and Cahn,^[^ ^56^ ^]^ in which the network (for instance, the host crystal lattice) provides an invariable reference frame for stress and strain and for interstitial diffusion.

The total free energy, $G^{S}$, of the system S is represented by a volume integral over a local Gibbs free energy density, Ψ (free energy per volume),

(1) $$G^{S}=\int_{S}\Psi dV$$

We take Ψ to depend on the temperature, *T*, the solute density, ρ, and on the stress (tensor) **S** as the state variables. Furthermore, we introduce the solute fraction, *x*, as the composition variable, and take ρ = *x*ρ_0_ with ρ_0_ a constant, the density of interstitial sites. Lagrangian variables, measuring all densities in coordinates of the undeformed state,^[^ ^57^ ^]^ are used throughout.

While we are interested in mapping temperature‐composition space, all variations and processes of interest to this study are isothermal. Thus, *T* may be treated as a label, and for conciseness its display is suppressed whenever appropriate. The fundamental equation for Ψ for isothermal processes is

(2) $$d\Psi =\mu \rho_{0}dx-E:dS$$

with the elastic strain (tensor) **E** and the solute chemical potential μ = (ρ_0_)^−1^dΨ/d*x*|_*T*, **S**_.

We take the system S as embedded in an external, fluid reservoir R which is at constant *T* and which loads the system boundary by a constant pressure, *P*_ext_. Note that internal heterogeneity can still create a more general stress state in S. Under closed‐system conditions, the net solute fraction $\bar{x}={\left(V^{S}\right)}^{-1}\int_{S}xdV$ in *S* is constant. At equilibrium subject to fixed values of *T* and *P*_ext_, the closed system S then minimizes its Gibbs free energy $G^{S}\left(T,x,P_{\mathrm{ext}}\right)$.

When analyzing open systems, we are also interested in processes in which solute is exchanged at equilibrium with R at controlled chemical potential. The thermodynamic potential that is minimized at equilibrium is then

(3) $$O^{S}=G^{S}-\mu N_{0}\bar{x}$$

the grand canonical potential.^[^ ^32^, ^58^, ^59^ ^]^

The description of heterogeneous systems also entails internal equilibrium conditions. Besides the established equilibrium conditions of continuum mechanics, it is required that the solute distributes so that

(4) μ=constant

even when there are gradients in stress.^[^ ^56^ ^]^

## 3 Model Scenario

In order to identify the most fundamentally relevant parameters and to clarify their impact, the analysis is based on the simplest materials model that yields the central behavior, namely a miscibility gap, elasticity and a coupling between chemistry and mechanics. To this end, we consider an interstitial regular solution with isotropic and (in the absence of diffusion) linear and composition‐independent elasticity and with linear and isotropic composition strain. The local stress in S will then affect the local chemical potential exclusively through the pressure, *P* = −1/3 Tr **S**.^[^ ^56^ ^]^ The composition‐strain coefficient, η, for linear and isotropic composition strain is defined so that the stress‐free lattice parameter, *a*, obeys *a* = *a*_0_ + η*x*.

The equation of state for the solute chemical potential is here (^[^ ^60^ ^]^ and see^[^ ^3^ ^]^ for the pressure dependence)

(5) $${\tilde{\mu }}_{P}\left(T,x,P\right)=\omega_{P}\left(1-2x\right)+RT\ln\frac{x}{1-x}+3\eta \Omega P$$

with *R* the gas constant and Ω ($=\rho_{0}^{-1}$) the volume per mole of interstitial sites. For conciseness of notation, Equation 5 takes the two‐phase coexistence at *P* = 0 as the reference state with $\tilde{\mu }=0$. The solute‐solute interaction energy parameter, ω_P_, at constant *P* is taken as positive‐valued, so that there is a miscibility gap below the critical temperature

(6) $$T_{P}^{C}=\frac{\omega_{P}}{2R}$$

**Figure 1:**
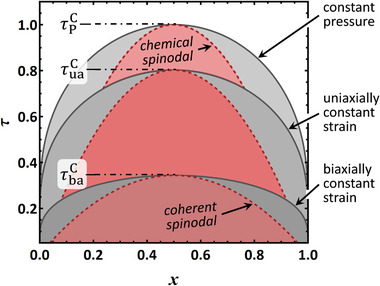
Binodals (solvus lines) and spinodals in equilibrium alloy phase diagrams of the regular solution in the domain of solute fraction, *x*, versus dimensionless temperature, τ.^[^ ^61^ ^]^ Boundary conditions: constant pressure, constant axial strain at uniaxial stress, and constant biaxial strain, as indicated by labels. Solid lines: binodals; dashed lines: spinodals. Labels for critical temperatures τ^C^ are displayed. Materials parameters motivated by Pd‐H, see Section 3.

The graph labeled ”constant pressure” in **Figure** **1** shows the binodals (solvus lines) of the well‐known equilibrium alloy phase diagram of the regular solution. The binodals are here obtained by solving $\tilde{\mu }\left(T,x,0\right)=0$ numerically for *x*.

For a regular solution with interaction energy parameter ω, the temperature, *T* ^S^, of the spinodals, where ∂μ/∂*x* = 0, is

(7) $$T^{S}\left(x,\omega \right)=\frac{2\;\omega }{R}x\left(1-x\right)$$

and the graph for ω = ω_P_ is the chemical spinodal in Figure 1. The figure's dimensionless temperature parameter is defined as $\tau =T/T_{P}^{C}$; the graphs are then independent of the ω_P_ magnitude.

For illustration, we match the materials parameter values to the Pd‐H system:^[^ ^62^, ^63^ ^]^ face‐centered cubic host lattice with lattice parameter $a_{0}=389.0\mathrm{pm}$,^[^ ^64^ ^]^ $T_{P}^{C}=566K$,^[^ ^65^, ^66^ ^]^ (polycrystalline) Young's modulus Y=120GPa, Poisson's ratio ν = 0.396,^[^ ^67^ ^]^ concentration‐strain coefficient η = 0.060.^[^ ^68^, ^69^ ^]^ Then, based on the $T_{P}^{C}$ value and Equation 6, $\omega_{P}\approx 9.41{\mathrm{kJ}\mathrm{mole}}^{-1}$.

## 4 Constrained Equilibrium Phase Diagrams

Our discussion of incoherent systems—which provide a benchmark—considers fluid‐like scenarios at constant pressure. For coherent systems, we also discuss scenarios where mechanical constraints fix the strain in one or two dimensions.

**Figure 2:**
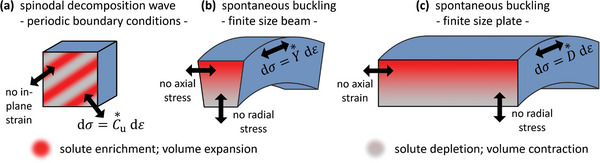
Schematic illustration of spontaneous chemo‐mechanically coupled modes in systems at constant overall composition and containing mobile solute. a) spinodal wave in a volume element from an infinitely extended solid or from a simulation with periodic boundary conditions. Strain is uniaxial, of magnitude, ε, along the wave vector; the conjugate stress component, σ, is determined by the uniaxial modulus ${\overset{*}{C}}_{u}$. b) Euler–Bernoulli type bending of a narrow beam, with full stress relaxation in radial and axial directions. Stress is uniaxial along the circumference, and the conjugate strain is determined by Young's modulus, $\overset{★}{Y}$. c) Kirchhoff type buckling of a laterally extended plate. Boundary conditions are no stress in radial direction and no strain in axial direction. The circumferential stresses and strains are coupled by the flexural modulus, $\overset{*}{D}$. Each mode may occur spontaneously when mobile solute leads to zero‐crossing in the respective, open‐system elastic parameter.

One example for the action of mechanical constraints is provided by the plane‐wave composition fluctuations of coherent spinodal decomposition in infinitely extended systems. The waves retain the in‐plane lattice parameter fixed at its stress‐free value.^[^ ^9^ ^]^ Thus, each wave has a uniaxial strain along the normal, from the composition modulation and ηδ*x*, and a biaxial in‐plane stress, see schematic in **Figure** **2**a. Spinodals and binodals may be derived, consistent with [9], based on displacement boundary conditions at fixed in‐plane strain (ref. [13], Sec VII 24 b).

A curved Euler–Bernoulli beam (Figure 2b) provides an example for a finite‐sized system in which the axial strain at each position is controlled by the curvature, while the stresses normal to the beam axis can relax, (ref. [70], Sec 88). The net stress is then uniaxial. Here again, the fixed axial strain results in a substantially modified equilibrium alloy phase diagram.^[^ ^71^ ^]^

The infinite plane waves of coherent spinodal decomposition exemplify a scenario that is compatible with the Bitter‐Crum theorem, whereas its finite size and the stress relaxation at the lateral surfaces make the beam incompatible with the theorem.

The discussion of the constant‐strain constraints of different dimensionality requires that *P* in Equation 5 be replaced with the appropriate strain‐dependent expression. Denoting by ε and σ a linear (projected on a given direction in space) strain or stress, respectively, we have

(8) $$\begin{array}{cc} P_{\mathrm{ua}} & =-\frac{1}{3}\sigma =-\frac{1}{3}Y\left(\varepsilon -\eta x\right) \end{array}$$

(9) $$\begin{array}{cc} P_{\mathrm{ba}} & =-\frac{2}{3}\sigma =-\frac{2}{3}\frac{Y}{1-\nu }\left(\varepsilon -\eta x\right) \end{array}$$

for uniaxially (beam) and biaxially (waves) controlled strain, respectively. Substituting *P* by Equations (8) or (9) introduces additional, *x*‐dependent terms in Equation 5, which can be combined with the *x*‐dependent solute‐solute interaction energy term to provide a rescaled effective interaction energy parameter,

(10) $$\omega_{\mathrm{eff}}=\omega_{P}-\frac{1}{2}3\eta \Omega \frac{\partial P}{\partial x}$$

Controlling the strain then provides equations of state $\tilde{\mu }\left(T,x,\varepsilon \right)$ (Equations S1 and S2, Supporting Information) in which ω is rescaled to

(11) $$\begin{array}{cc} \omega_{\mathrm{ua}} & =\omega_{P}-\frac{1}{2}Y\eta^{2}\Omega \end{array}$$

(12) $$\begin{array}{cc} \omega_{\mathrm{ba}} & =\omega_{P}-\frac{Y}{1-\nu }\eta^{2}\Omega \end{array}$$

As all factors in the correction terms are regularly positive, the mechanical constraints will invariably reduce the solute‐solute interaction energy. The reduction is analogous to what has been inferred for alloying at constant volume.^[^ ^3^ ^]^

By Equation (6), the reduced interaction energies lead to reduced critical temperatures for the constrained systems:

(13) $$\begin{array}{cc} T_{\mathrm{ua}}^{C} & =T_{P}^{C}-\frac{Y\eta^{2}\Omega }{4R} \end{array}$$

(14) $$\begin{array}{cc} T_{\mathrm{ba}}^{C} & =T_{P}^{C}-\frac{Y\eta^{2}\Omega }{2(1-\nu )R} \end{array}$$

where $T_{\mathrm{ba}}^{C}$ agrees with the critical temperature of the coherent spinodal.^[^ ^9^ ^]^

With the Pd‐H‐inspired materials parameters, the values of the dimensionless temperature at criticality in the constrained systems emerge as $\tau_{\mathrm{ua}}^{C}=0.802$ and $\tau_{\mathrm{ba}}^{C}=0.344$, see Figure 1. Note, that the equation of state for μ(*x*) remains otherwise that of a conventional regular solution. Thus, the equilibrium alloy phase diagrams of the constrained systems emerge as conventional regular solution alloy phase diagrams, just with different critical temperatures. Figure 1 displays the three phase diagrams, incoherent and with axially or biaxially constant strain.

Biaxially constant strain is a boundary condition when thin metal films are hydrided while clamped to a rigid substrate, or when epitaxial semiconductor layers form concentration patterns by coherent spinodal decomposition. Reports of narrowed miscibility gaps and of suppressed critical temperatures under those conditions are qualitatively consistent with Figure 1.^[^ ^72^, ^73^, ^74^ ^]^ Furthermore, theory and experiment agree that mechanical constraints by rigid interfaces reduce ω in hydride nanoparticles.^[^ ^51^, ^53^, ^55^, ^75^ ^]^

Additional phenomena are observed when the (conventional, constant‐composition) closed‐system elastic parameters of clamped thin films depend on the composition. Epitaxy stresses then give rise to a dielastic interaction energy with heterogeneities in the local stiffness that may induce coherent spinodal spilling outside the chemical spinodal.^[^ ^44^ ^]^

## 5 Open‐System Mechanical Stability Analysis

### 5.1 Open‐System Elastic Parameters

We now turn to inspecting the inherent elastic stability of initially homogeneous systems containing mobile solute. For the isotropic system at constant *x* (no diffusion), the elastic response can be parameterized by Young's modulus, *Y*, and Poisson's ratio, ν. In solids containing a mobile solute species, equilibrium is at uniform chemical potential, and the elastic parameters are then stress‐strain derivatives at constant μ.^[^ ^56^ ^]^ These open‐system equivalents to *Y* and ν are^[^ ^56^ ^]^

(15) $$\begin{array}{ccc} \overset{★}{Y} & = & \frac{Y}{1+\chi Y\eta^{2}} \end{array}$$

(16) $$\begin{array}{ccc} \overset{★}{\nu } & = & \frac{\nu -\chi Y\eta^{2}}{1+\chi Y\eta^{2}} \end{array}$$

with χ a solute susceptibility parameter,

(17) $$\chi =\Omega \frac{dx}{d\mu }{|}_{T,P}$$

For the regular solution (see Equation 5), χ emerges as

(18) $$\chi \left(T,x,\omega \right)=\frac{x(1-x)\Omega }{RT-2x(1-x)\omega }$$

independent of the pressure.

All other elastic parameters of the isotropic solid can be derived from *Y* and ν, and this applies also to the respective open‐system parameters. Specifically,

(19)

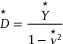

is the open‐system flexural modulus, an axial stress–strain derivative at constant stress in one orthogonal direction and constant strain in the other. Furthermore,

(20)

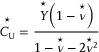

is the open‐system's uniaxial modulus, an axial stress–strain derivative at constant strain in both orthogonal directions.

**Figure 3:**
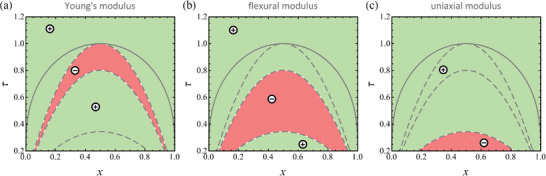
Mapping the signs of open‐system elastic parameters in the domain of solid‐fraction, *x*, and dimensionless temperature, τ.^[^ ^61^ ^]^ a) Young's modulus $\overset{★}{Y}$, Equation (15); b) flexural modulus $\overset{★}{D}$, Equation (19); c) longitudinal modulus $\overset{★}{C_{U}}$, Equation (20). Green, positive; red, negative. Solid line: constant‐pressure (incoherent) binodal; dashed lines: spinodals, as in Figure 1 (from top to bottom: chemical, uniaxial‐strain, and coherent spinodal). Note that each sign inversion coincides with one of the spinodals of Figure 1. Regions of negative elastic parameters can be linked to unstable chemo‐mechanically coupled modes. Materials parameters motivated by Pd‐H, see Section 3.

At the chemical spinodal, where dμ/d*x*|_*T*, *P*_ = 0, Equation (17) implies a pole in χ, which can entail a sign inversion of the elastic parameters.^[^ ^76^, ^77^ ^]^ Regions of temperature‐composition space with negative elastic parameters, as they emerge from Equations ((15), (16), (17), (18), (19), (20)), are illustrated in **Figure** **3**. Note that each sign inversion coincides with one of the spinodal lines of Figure 1.

### 5.2 Elastic Instability

Stiffness parameters such as $\overset{★}{Y}$, $\overset{★}{D}$ or $\overset{★}{C_{U}}$ measure restoring forces that counteract elastic strain about the stress‐free state. Stability requires those parameters to be positive‐valued, since otherwise a small deformation will set up stress acting to further amplify the deformation. Thus, the stress‐free state is unstable in an elastic medium of negative stiffness, the medium will spontaneously distort. Spontaneous distortion as a consequence of open‐system elasticity was first pointed out in ref. [78] and has since been confirmed in ref. [15, 71]. More specifically, a solid is unstable against spontaneous distortion in any specific mode of deformation that does work against a negative‐valued elastic parameter. The nature of such modes must depend on the shape of the system and on the mechanical boundary conditions.

We hypothesize that, for any given sample geometry, there is a finite number of specific modes for decomposition at constant net solute content. We take each mode to be characterized by the strain field E(r)=Ae(r) with A the amplitude and **e**(**r**) a basis strain field that depends on the position, **r**. **E** must satisfy the compatibility conditions of mechanics, in other words, the strain must follow as the derivative of a displacement vector field, (ref. [70], Sec 9.77). For compatibility with constant net solute content, the mode must also conserve the net volume.

The mechanical work *W* associated with any given mode assumes an extremum for A=0. For a small deformaton around the stress‐free state (small A), *W* is a convex function of A in stable states but a concave function of A in unstable states. In a linear stability analysis, the instability criterion is then

(21) $$\frac{d^{2}W}{dA^{2}}{|}_{A=0}=0$$

As the amplitude is increased starting from 0, the system‐integrated mechanical work can be computed as

(22) $$W=\int_{S}\int_{0}^{A}S:\frac{dE}{dA}dA\;dV=\int_{S}\int_{0}^{A}S:e\;dA\;dV$$

We then have

(23) $$\frac{dW}{dA}{|}_{A=0}=\int_{S}S:e\;dV$$

where the stress is $S=\overset{★}{C}E=\overset{★}{C}eA$ with $\overset{★}{C}$ the open‐system stiffness tensor. In view of Equation 23, the instability criterion of Equation (21) is then implied as as

(24) $$0=\frac{d^{2}W}{dA^{2}}{|}_{A=0}=\int_{S}\frac{dS}{dA}:e\;dV=\int_{S}\left(\overset{★}{C}\;e\right):e\;dV$$

For isotropic elasticity, the expression on the right‐hand side of that equation depends on the elastic response by a combination of the elastic parameters in Section 5.1. Since **e** is a constant, evaluating the instability criterion amounts to finding combinations of *T* and *x* (or μ) for which that combination changes sign.

With attention to the examples of Section 4, the strain field in a curved Euler‐Bernoulli beam does work against Young's modulus, and the plane‐wave fluctuations of spinodal decomposition do work against the uniaxial modulus. Figure 3 confirms that the regions of elastic instability differ between those parameters.

### 5.3 Instability Toward the Spontaneous Formation of Composition Gradients

The coupled chemical and mechanical equilibrium in a solid solution may be derived by considering the mechanical equilibrium alone, accounting for the (generally nonlinear^[^ ^76^ ^]^) open‐system elastic response.^[^ ^56^ ^]^ The composition field then follows from the stress/strain‐field, along with the equations of state and with the acting values of μ and *T*.^[^ ^56^, ^79^ ^]^ This implies that the open‐system elastic instabilities have a one‐to‐one match to instability towards the spontaneous formation of composition gradients. In other words, some scenarios afford the analysis of chemical stability to be based entirely on a linear stability analysis of the mechanical equilibrium.

By means of example, consider the plane wave fluctuations of coherent spinodal decomposition in a large (negligible surface effects) system S, Figure 2(a). As explained in Section 4, each wave has a uniaxial strain along the direction of its wavevector, **q**. Let **u** be a unit vector along that direction. The basis strain field is **e** = **u**⊗**u** sin (**q** · **r**), with ⊗ the outer product operator. The kernel of the integral in Equation 24 is then readily evaluated using Hooke's law for isotropic elasticity. In terms of the open‐system elastic parameters one obtains $\left(\overset{★}{C}\;e\right):e=1/2\;\overset{★}{C_{U}}\sin^{2}\left(q\cdot r\right)$. Integrating the sin ^2^‐term is also straightforward, and the instability criterion emerges as

(25) $$0=\frac{1}{4}\;\overset{★}{C_{U}}V^{S}$$

where $V^{S}$ denotes the volume of S. The criterion Equation 25 can only be fulfilled if $\overset{★}{C_{U}}=0$. Exploiting the equations of Section 5.1 for the open‐system elastic parameters, one readily finds that sign change precisely along the coherent spinodal, which is Equation 7 with ω = ω_ba_ (see Equation 12). The location of the sign‐change of $\overset{★}{C_{U}}$ in Figure 3c) illustrates this finding. The mechanical linear stability analysis correctly predicts that coherent composition waves in system with negligible surface effects become unstable at the coherent spinodal, which is the well‐established instability line for this problem. This exemplifies and supports the mechanical stability analysis using open‐system elasticity as an approach towards identifying chemical instability.

Note that the mechanical stability analysis for the uniform, stress‐free system can be based on the linear, small‐stress open‐system elastic parameters. The mechanics of the final equilibrium state of unstable systems will in general be governed by higher‐order elastic parameters.

## 6 Transformation Mechanism Maps

### 6.1 Incoherent System

We now turn to inspecting phase transformations and their mechanisms in temperature‐composition space. The results are conveniently communicated by means of transformation mechanism maps that document whether and how a system transforms under the premise that it has been brought to the respective values of *T* and *x* in an initial, uniform state.

**Figure 4:**
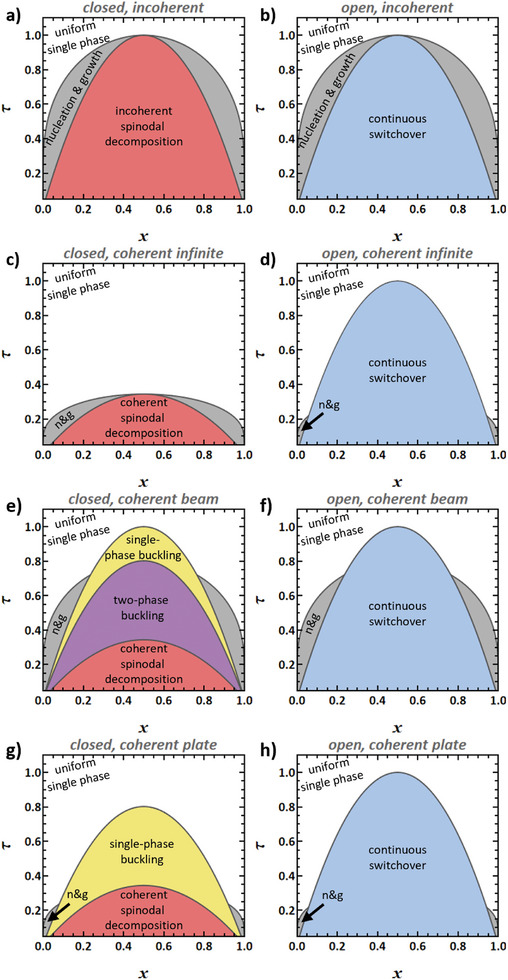
Transformation mechanism maps in the domain of solid‐fraction, *x*, and dimensionless temperature, τ^[^ ^61^ ^]^ for closed (left column) and open (right column) systems with a miscibility gap. a,b) Incoherent phase transformation. c,d) Infinite coherent system obeying the Bitter‐Crum theorem. e,f) beam‐shaped, finite‐size coherent system. g,h) Plate‐shaped, finite‐size coherent system. Note the several fundamentally different transformation pathways, as indicated by labels and differently colored shading. Note also that all diagrams are for the identical set of materials parameters, in other words, for the identical binary alloy system. It is only the boundary conditions and the sample shape that change. Materials parameters motivated by Pd‐H, see Section 3.

In the closed, incoherent system, the overall amount of solute is constant and phase transformations are at constant and uniform pressure. This is the textbook case and the transformation mechanism map of **Figure** **4**a holds no surprises. Right inside the binodal, the uniform solution is metastable and can transform by nucleation and growth (gray area). Closed systems are unstable with respect to spontaneous decomposition when quenched into the regime (red region in Figure 4a) of spinodal instability.^[^ ^9^ ^]^ This regime is delimited by the chemical spinodals (dashed lines).

**Figure 5:**
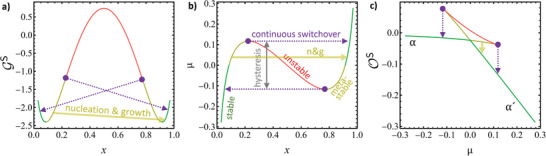
Various ways of displaying the free energy variation of the open, regular solution system at constant pressure. a) Total free energy $G^{S}$ versus solute fraction *x*. b) Solute chemical potential μ versus *x*. c) Grand canonical potential $O^{S}=G^{S}-\mu N_{0}x$ versus μ. Green segments of graph: stable states in dilute (α) and concentrated (α′) phase; yellow: metastable states; red: unstable state. Broad yellow arrows: nucleation and growth of the concentrated phase. Dashed violet arrows: continuous switchover process, triggered at a spinodal point. Hysteresis in μ for forward and backward switchover is indicated. Example for the regular solution of Section 3, with materials parameters motivated by Pd‐H and at dimensionless temperature τ = 0.7. Ordinates use arbitrary units.

In the open, incoherent system, solute is exchanged with an external reservoir R that maintains the chemical potential at a constant value $\mu^{R}$. **Figure** **5** shows free energy representations – color‐coded for stable, metastable, and unstable states – for the system, S, at an exemplary τ. As was mentioned above, the Gibbs free energy, $G^{S}$, is minimized at equilibrium in the closed system, whereas the grand canonical potential of Equation (3) is minimized in the open system. Yellow arrows in the figure illustrate the well‐known fact that nucleation and growth can act for the same concentrations as in the closed system. The gray region in Figure 4b represents this process.

It is well known that nucleation can be retarded or even entirely suppressed in open interstitial solutions. Wagner^[^ ^50^ ^]^ has first pointed out that metastable states of the dilute phase are then possible for chemical potentials $\mu^{R}$ extending up to that of the spinodal, as denoted by the yellow segment of the α‐phase part of the free energy graph in Figure 5b. When $\mu^{R}$ exceeds the chemical potential value at the spinodal (red segments of graphs in Figure 5), then the solution can no longer be at equilibrium with the reservoir, and there is no barrier for further uptake of solute. This process can continuously increase *x* and it does not involve the formation of a distinct new phase. Depending on the kinetics, the switchover to the new phase may even take the form of a uniform alloying of the entire system. The continuous composition evolution prevails until the alloy reaches a new equilibrium that is completely in the stable concentrated phase, as illustrated by dashed arrows in Figure 5.

During the backwards transformation, the analogous process starts at the opposing spinodal; this results in hysteresis, as indicated in Figure 5b. A survey of Pd nanoparticles confirms that phase‐transformation hysteresis in some studies indeed extends up to the full spinodal spread.^[^ ^55^ ^]^ While this reasoning emphasizes the relevance of the chemical spinodal, it is also emphasized that hysteresis in experimental scenarios may have a different origin. In classic, incoherent transformation pathways two phases coexist and the interface sweeps the system as the phase fraction changes. Observations of hysteresis are then compatible with sluggish interface motion through a landscape of pinning sites at crystal lattice defects and specifically at misfit dislocations from previous phase transformations.^[^ ^32^ ^]^

Arrows in Figure 5c illustrate that the just‐mentioned process will indeed decrease $O^{S}$. This confirms that, beyond the spinodal, the open system can transform by continuously—without phase transformation and, hence, without nucleation barrier—evolving in composition. This continuous switchover instability is represented by the blue region in the transformation mechanism map of Figure 4b.

Continuous switchover in Pd‐H is observed in a numerical study using periodic boundary conditions,^[^ ^22^ ^]^ and in‐situ transmission electron microscopy studies of phase‐change in Pd‐H observe no two‐phase intermediate states in nanoparticles^[^ ^53^ ^]^ and in nanorods when below a critical size.^[^ ^47^ ^]^ In the context of battery electrodes, continuous switchover is observed during lithiation of ${\mathrm{Li}}_{x}{\mathrm{FePO}}_{4}$ (see refs. [8, 37] and Section 1). For incoherent systems, the analysis in [50] clarifies that this process must be expected at the chemical spinodal.

### 6.2 Infinitely Extended Coherent System

We now turn to a coherent and infinitely extended system. In the sense of a stability analysis, small composition modulations can here be Fourier transformed and stability then discussed with reference to the plane waves of coherent spinodal decomposition. As stated in Section 4, the discussion is then compatible with the Bitter‐Crum theorem.

Regarding the closed system, the considerations in Section 4 on coherent spinodal decomposition imply immediately that the transformation mechanism map (Figure 4c) here follows the biaxially controlled‐strain phase diagram in Figure 1. Note that nucleation—in the gray nucleation‐and‐growth regions of the figure—is here coherent. The underlying equation of state accounts for the in‐plane coherency constraints, while the out‐of‐plane boundary condition is stress‐free. Thus, no extra coherency strain energy is involved. The nucleation barrier is then a classic barrier, governed by (for instance, Cahn‐Hilliard‐type) interfacial energy. This barrier may be overcome by thermal activation in practical scenarios. The unsurmountable macroscopic barrier to nucleation of [28] is not encountered in the nucleation‐and‐growth region of the biaxally‐controlled‐strain phase diagram. However, note the extended interval of temperature between $T_{\mathrm{ba}}^{C}$ and $T_{P}^{C}$, in which the infinite (Bitter‐Crum type) closed coherent system does not transform since the single‐phase state is stable at equilibrium.

The gray nucleation‐and‐growth regions in Figure 4d reflect that all transformations of the closed system may again be found in the corresponding open system. Yet, additional instabilities, specific to the open system, may intervene. This applies here to continuous switchover. As this can be a uniform change, not involving composition gradients and so without coherency strain energy, it must be expected to act in the same way in open coherent as in open incoherent systems. In other words, the onset of continuous switchover is expected at the chemical spinodal. That is illustrated by the blue region in Figure 4d.

Somewhat counterintuitively, the regions of instability differ drastically between transformation mechanism maps for the closed and the open coherent system. This is confirmed by an independent inspection of the free energy of Bitter‐Crum type systems in Section S2 (Supporting Information). That section also emphasizes the macroscopic energy barrier of [28], which prohibits thermally activated nucleation outside of the nucleation‐and‐growth region of the biaxally‐controlled‐strain phase diagram.

### 6.3 Finite‐Size Beam‐Shaped Coherent System

Next, we discuss the beam geometry of Section 4 and Figure 2b, exemplifying that relaxation at free surfaces may lead to deviations from the Bitter‐Crum theorem.

According to Section 4, the conditions for two‐phase equilibrium in the closed system are here governed by the constant‐axial‐strain phase diagram of Figure 1, of which the gray regions in Figure 4e represent the nucleation‐and‐growth regime. As a region of new phase nucleates in the initially homogeneous and straight beam, subsequent growth may involve radial solute redistribution for energy minimization—the phase with the larger lattice parameter may relocate to the tensile fiber at one side of the beam, which will simultaneously bend.

Regions of instability are superimposed on the above diagram. The beam has a chemo‐elastically coupled bending mode with mechanical work against $\overset{★}{Y}$. That parameter changes sign at the chemical spinodal (Figure 3a). In view of Section 5, this implies an elastic instability with the spontaneous formation of buckled states at no load (yellow region in Figure 4e).

**Figure 6:**
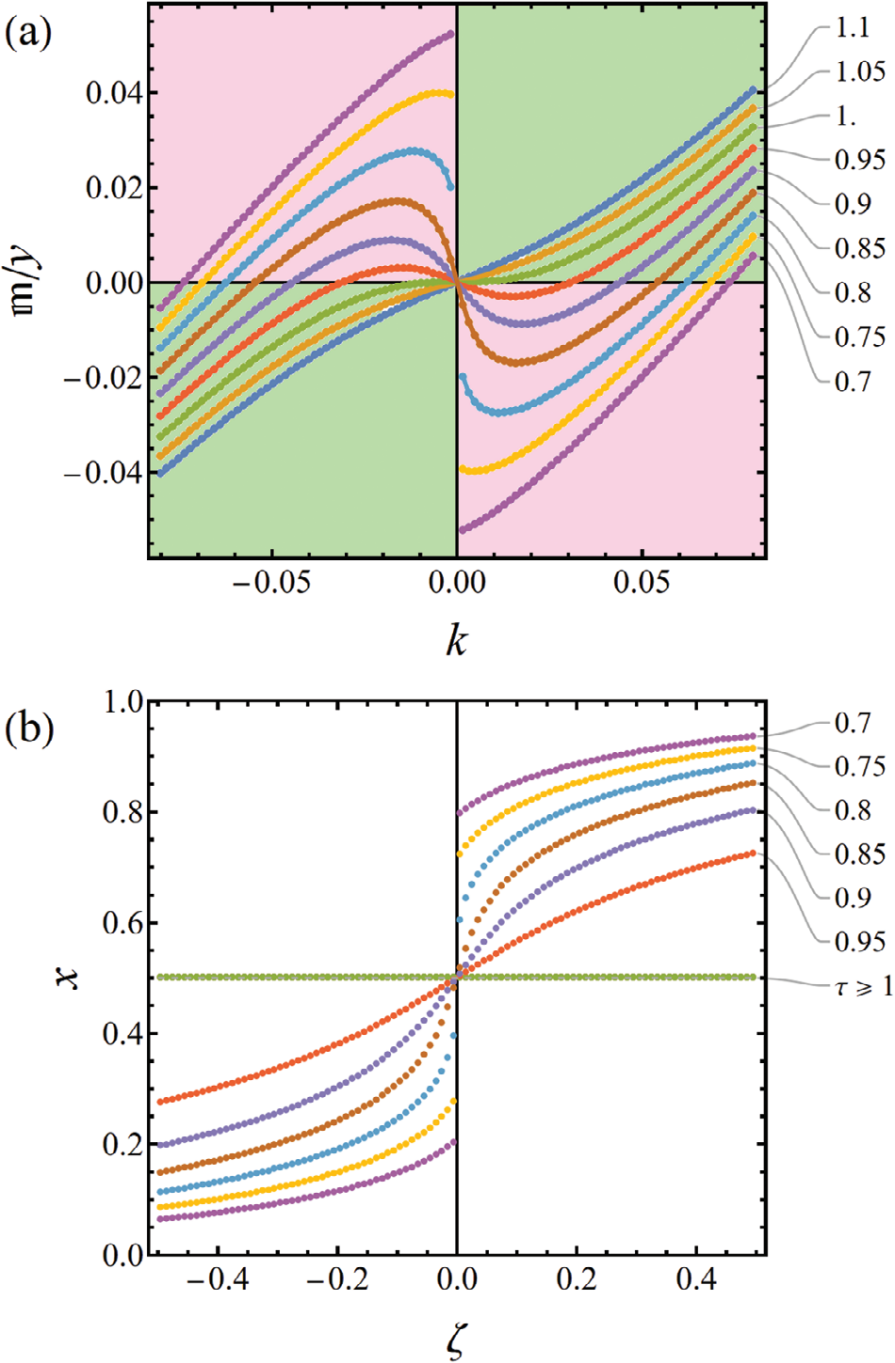
Chemo‐elastically coupled modes in a closed coherent system. Results for bending instability in a long, thin beam free of external load, at mean solute fraction *x* = 0.5 and at different temperatures (τ = 0.70 to 1.10 in steps of 0.05, see labels). a) Dimensionless bending moment m versus dimensionless curvature *k*.^[^ ^61^ ^]^ Red shaded regions denote instability against spontaneous bending, green stability. Zero‐crossings with positive slope represent equilibrium states. b) Concentration profile of solute fraction *x* versus dimensionless *z*‐coordinate ζ^[^ ^61^ ^]^ for the equilibrium states. In both panels, note continuous variation through *k* = 0 or ζ = 0, respectively, when τ ⩾ 0.80, discontinuity when τ < 0.80. This corresponds to transition from single‐phase to dual‐phase structure at equilibrium. Materials parameters motivated by Pd‐H, see Section 3.

We have applied the procedures of [71] (see Section S4, Supporting Information, for details) for investigating the bending modes. In brief, a continuum analysis finds the equilibrium composition field in a beam of prescribed curvature and fixed overall composition, subject to the condition of uniform chemical potential. The stress in each fiber is obtained by inverting Equation (5), thereby accounting for the full nonlinear elastic stress‐strain response. **Figure** **6**a displays exemplary results for the dimensionless bending moment^[^ ^61^ ^]^ m versus the dimensionless curvature^[^ ^61^ ^]^ *k* in a beam of net solute fraction $\bar{x}=0.5$ for different dimensionless temperatures τ. For τ ⩾ 1, the moment‐curvature relations occupy the first and third quadrants, which correspond to stability of the straight beam. By contrast, the graphs for τ < 1 contain segments in the second and fourth quadrants that correspond to mechanical instability. Each of those graphs has stable states (m=0) at finite curvature values, confirming spontaneous buckling after quenching into the regime of (chemical) spinodal instability.

Figure 6b shows the radial composition profiles of the above stable states. The profiles are smooth for τ > 0.8, indicating a single‐phase state with a continuous variation in composition. Discontinuities indicate two‐phase states at τ < 0.8. Those latter states may be inferred independently from the region of spinodal instability in the constant‐axial‐strain phase diagram, the violet region in Figure 4c. It represents an instability against the formation of a dual‐phase structure by the continuous growth of initially small, radial composition fluctuations, with no need for nucleation. The numerical analysis and the phase diagram here agree, supporting both approaches.

Any system will be unstable with respect to decomposition when in the coherent spinodal region, shown in red in Figure 4c. Plane composition waves can here grow in amplitude. As discussed in Section 5.3, the coherent spinodal instability is naturally compatible with a linear stability analysis based on open‐system mechanics.

Regarding the open system, we first observe that continuous switchover at the chemical spinodal is again expected to act as in an incoherent system. This is illustrated by the blue region in the transformation mechanism map of Figure 4f.

The continuous switchover instability in open coherent systems is consistent with the thermodynamic hysteresis of [28], reflecting the difference between the chemical potentials at the spinodals, Figure 5b. Yet, as all transformations of the closed system may also occur in the open one, the nucleation‐and‐growth regime of the constant‐axial‐strain phase diagram of Figure 1 pertains (gray regions in Figure 4f). At not too high temperature, coherent nucleation may then act before the chemical potential reaches the spinodal, diminishing the hysteresis. In support of this conclusion, nanoporous Pd‐H with beam‐like microstructural elements (“ligaments”) was found stable for ≫1000 transformation cycles, suggesting a coherent phase change,^[^ ^69^ ^]^ while its charging‐rate dependent hysteresis^[^ ^77^ ^]^ suggests nucleation.

For verification, Section S3 (Supporting Information) explores the criterion by Schwarz and Khachaturyan^[^ ^28^ ^]^ for instability against coherent precipitation, applied to the beam scenario. The instability is found precisely at the binodal of the constant‐axial‐strain phase diagram and so coincides with the nucleation‐and‐growth region in Figure 4f. Thus, the comparison provides independent support for our transformation mechanism map of the open coherent system. The coincidence of the instability of [28] with the present work's onset of nucleation is a natural consequence of the neglect of interfacial energies in both approaches; this renders the two mechanisms indistinguishable in our analysis.

Chemo‐elastically coupled buckling modes at no load emerge also from theory of diffusive phase change^[^ ^15^, ^78^ ^]^ and of equilibrium in closed coherent systems.^[^ ^71^ ^]^ Buckling during lithiation of silicon nanowires^[^ ^18^ ^]^ and hoyneycombs^[^ ^29^ ^]^ provides a direct experimental confirmation, yet for a transformation that may include crystallographic changes. For nanoporous Pd‐H—as a solid solution—a large and transient contraction is reported when the net hydrogen fraction moves into the miscibility gap.^[^ ^80^ ^]^ The explanation in terms of hydrogen‐induced plasticity^[^ ^80^ ^]^ may be challenged in view of the transient nature of the phenomenon. The spontaneous buckling of the ligaments would naturally explain the observation.

### 6.4 Finite‐Sized Plate‐Shaped Coherent System

As a further geometry, consider a thin, laterally extended plate. Cylindrical bending provides a possible deformation mode, as illustrated in Figure 2c. For the radial and axial directions, Kirchhoff plate theory implies no stress and no strain, respectively, whereas a bending strain acts circumferentially and works against the flexural modulus, *D* = *Y*/(1 − ν^2^) (ref. [81], Sec 7.6). As the strain is controlled in two orthogonal directions, two‐phase coherent coexistence follows the phase diagram for biaxially constant strain of Figure 1. The gray nucleation‐and‐growth regions and the coherent spinodal region in Figure 4g represent that phase diagram.

In analogy to the beam geometry, a single‐phase buckling instability results from the sign‐change in the relevant open‐system elastic parameter, namely $\overset{*}{D}$. That parameter is negative between the uniaxially‐controlled‐strain spinodal and the coherent spinodal (Figure 3), which is the yellow region in Figure 4g.

The analysis of phase change behavior in the open, coherent, plate‐shaped system follows the same line of reasoning as for the beam geometry. Nucleation and growth as well as coherent spinodal decomposition are allowed in the two‐phase and spinodal regions of the biaxially constant‐strain phase diagram, respectively. A region of continuous switchover is again superimposed, see Figure 4h.

As a remarkable observation, the temperature range $\tau_{\mathrm{ua}}^{C}$ (≈0.8) to 1 in the transformation mechanism maps of Figure 4g,h has unlimited solubility at equilibrium for the closed system, yet a continuous switchover instability for the open one. In this range, if the solute uptake were stopped partway through the switchover, the system would convert to a closed one and the composition field would homogenize to account for the new equilibrium state.

## 7 Capillarity and Size Dependence

### 7.1 Effectively Inert Surface

As an example for the impact of capillarity, we consider a plate with superficial layers that retain sensibly constant composition while the bulk is subject to the composition change and/or strain of a phase transformation. The layers then deform by conventional, constant‐composition elasticity and so are substantially stiffer than the bulk. This is expected when the surface region dissolves no solute or, alternatively, when there is a large negative enthalpy of segregation, so that the surface saturates in solute at a chemical potential substantially below that of the bulk phase transformation.

Superficial solute segregation or depletion is established in lithium iron phosphate, where facets with specific crystallographic orientation tend to be either fully lithiated or delithiated, see ref. [35] and Section 7.4 below. For Pd‐H, the early saturation of a thin superficial layer is well supported by observations on H underpotential deposition.^[^ ^82^ ^]^ Studies of Pd‐H nanoparticles^[^ ^53^, ^55^ ^]^ and nanocrystalline materials^[^ ^75^, ^83^, ^84^ ^]^ have confirmed that the surface or internal interfaces here act effectively as a stiffening confinement, opposing the bulk phase transformation and narrowing the miscibility‐gap or reducing its critical temperature.^[^ ^51^ ^]^

### 7.2 Stress Balance and Spinodals

**Figure 7:**
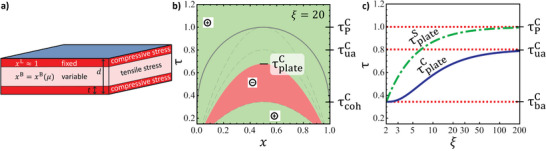
Chemo‐mechanically coupled instability in a plate‐shaped specimen with coherent inert surface layers. a), schematic illustration of the initial geometry, straight plate with in‐plane coherency stresses of opposite sign in bulk and layers. b), color‐coded map of the sign of the effective flexural modulus *D*_eff_ (Equation 32) in the domain of reduced temperature τ and bulk solute fraction *x*. Green, positive; red, negative. Example for reduced (normalized to inert layer thickness^[^ ^61^ ^]^) plate thickness ξ = 20. Reduced critical temperatures of the chemical and coherent spinodal are labelled as $\tau_{P}^{C}$ and $\tau_{\mathrm{coh}}^{C}$, respectively. Dashed lines (from top to bottom): chemical spinodal, uniaxial constant strain spinodal, coherent spinodal. Solid line: binodal. Reduced upper consolute temperature for the buckling instability is marked as $\tau_{\mathrm{plate}}^{C}$. c) Log‐linear representation of $\tau_{\mathrm{plate}}^{C}$ (blue solid line) versus ξ, Equation (33). Green dash‐dotted line: critical temperature, $\tau_{\mathrm{plate}}^{S}$, for the continuous switchover instability. Materials parameters motivated by Pd‐H, see Section 3.

We consider an initially planar plate of thickness *d* in the absence of external load. The bulk region B of thickness *d* − 2*t* is bordered, near each surface, by a superficial layer L of thickness *t*, see **Figure** **7**a. The solute fraction, $x^{B}$, in B equilibrates at constant μ according to the regular solution equation of state, Equation (5), whereas the solute fraction in L is fixed at the constant value $x^{L}$.

Layers and bulk are considered coherent, with uniform and isotropic in‐plane strain ε_‖_ throughout the plate. The plate is stress‐free in the normal direction, and the in‐plane stress component in each region is σ_‖_ = *B*(ε_‖_ − η*x*) with *x* the local solute fraction, η*x* the local stress‐free strain, and *B* = *Y*/(1 − ν) the biaxial modulus. At equilibrium, the volumetric mean of the σ_‖_ must vanish, and this is readily found (Section S5, Supporting Information) to imply

(26) $$\sigma_{∥}^{B}=\frac{Y\eta }{1-\nu }\;\frac{2}{\xi }\left(x^{L}-x^{B}\right)$$

for the in‐plane stress in the bulk. Here, ξ denotes a dimensionless plate thickness parameter, ξ = *d*/*t*. If solute that expands crystal lattice (η > 0) is enriched at the surface, then the in‐plane stress is tensile in the bulk and compressive in the surface. In a 2D representation of the surface, the stresses in layers and bulk would be represented by surface stress and surface‐induced bulk stress, respectively, and their balance would be expressed by the generalized capillary equation for solids.^[^ ^85^ ^]^

According to Equation (5) and since *P* = −2/3 σ_‖_, the surface‐induced stress of Equation (26) shifts the solute chemical potential in the bulk, relative to a reference system at same composition and temperature and with no capillarity, by

(27) $$\Delta \mu^{B}=3\eta \Omega P=\frac{Y\eta^{2}\Omega }{1-\nu }\;\frac{4}{\xi }\left(x^{B}-x^{L}\right)$$

As $x^{L}$ is a constant, $\Delta \mu^{B}$ varies linearly with the bulk composition, and Equation (10) implies the rescaled solute–solute interaction energy

(28) $$\omega_{\mathrm{plate}}=\omega_{P}-\frac{Y\eta^{2}\Omega }{1-\nu }\;\frac{2}{\xi }$$

It is seen that the inert surface layers act as a mechanical confinement; this is analogous to the constraints of fixed strain in Section 4, except that the severity of the constraint is here size‐dependent.

The rescaling of the interaction energy is again reflected in a shift of the spinodal and, hence, of the instability line for continuous switchover. In view of Equation (7), the instability is here found at

(29) $$T^{I}=4\;T_{\mathrm{plate}}^{S}\;x^{B}\left(1-x^{B}\right)$$

with

(30) $$T_{\mathrm{plate}}^{S}=T_{P}^{C}-\frac{Y\eta^{2}\Omega }{2R(1-\nu )}\;\frac{2}{\xi }$$

The dash‐dotted line in Figure 7c illustrates the variation of $T_{\mathrm{plate}}^{S}$ with ξ.

**Figure 8:**
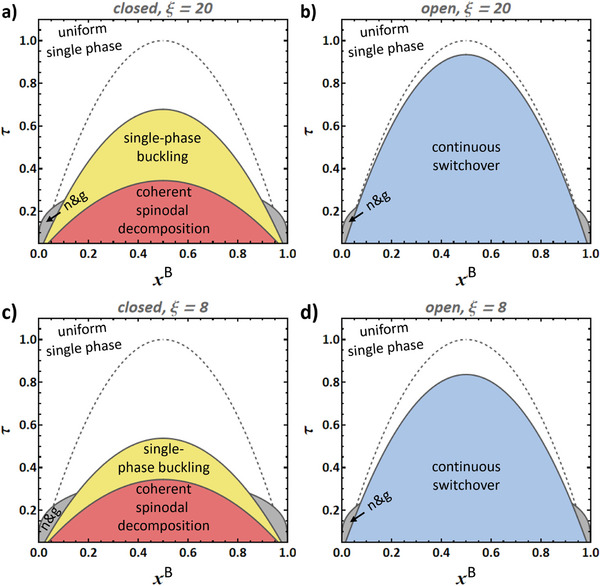
Transformation mechanism maps in the domain of bulk solid fraction, $x^{B}$, and dimensionless temperature, τ,^[^ ^61^ ^]^ here for the plate‐shaped system with an inert surface layer. Top and bottom rows, plates of reduced thickness (normalized to thickness of the inert layer^[^ ^61^ ^]^) ξ = 20 and 8, respectively. Left column, closed system; right column, open system. Yellow, red and blue regions denote instabilities of the initially uniform system, as labeled. Gray regions: nucleation and growth. Dashed line: chemical spinodal, shown as reference. Note that regions of single‐phase buckling instability and of continual switchover instability depend individually and differently on the plate thickness. Materials parameters motivated by Pd‐H, see Section 3.

Exemplary transformation mechanism maps for two different thickness values in **Figure** **8** advertise that the continuous switchover is here expected in a more restricted region, inside of the chemical spinodal and progressively suppressed at reduced plate thickness. Note that the maps of Figure 8 use $x^{B}$ as the composition parameter. If plotted versus the net composition, including the enriched or depleted surface layer, the instability lines would be asymmetrically shifted against the composition axis.

### 7.3 Cylindrical Buckling Instability

Next, we inspect the stability with respect to cylindrical buckling. As the stresses of layer and bulk in the straight plate are internally equilibrated, and as our linear stability analysis is restricted to small strain and linear elasticity, the superposition principle of mechanics implies that the bending strains do no work against the preexisting stresses. Thus, the approach of Section 5.2 applies and Equation (24) supplies the criterion of instability.

With **u** a circumferential unit vector, tangential to the curvature (of magnitude κ) and with *z* a radial position variable with the origin in the centre plane of the plate, we may take **e** = (*z*/*d*) **u**⊗**u** as the basis strain field (Section 5.2) for the cylindrical bending, and A=κd as the deformation amplitude parameter. Here again, mechanical work is done exclusively by the circumferential bending strain, which is ε = κ*z*. Thus, the mechanical free energy density, Ψ_mech_, for bending depends on ε and on the circumferential bending stress component σ as Ψ_mech_ = 1/2σε. Contrary to the uniform plate of Section 6.4, the elastic coefficients differ, namely the constant‐composition flexural modulus *D* in the layers and the open‐system flexural modulus $\overset{*}{D}$ in the bulk. Accounting for this distinction, the net mechanical work of bending deformation for a plate of thickness *d* and area *A* is obtained as

(31) $$W=A\int_{-d/2}^{d/2}\Psi_{\mathrm{mech}}dz=\frac{1}{24}Ad^{3}\kappa^{2}D_{\mathrm{eff}}$$

Here, *D*_eff_ is an effective flexural modulus for the plate,

(32) $$D_{\mathrm{eff}}=\overset{★}{D}+\left(D-\overset{★}{D}\right)\frac{6\xi^{2}-12\xi +8}{\xi^{3}}$$

The ξ‐dependence of *D*_eff_ reflects the action of the two different elastic parameters emerges from carrying the integration in Equation (31) over the two regions, bulk and layers (see Section S5, Supporting Information), where the elastic parameters differ.

In view of Equation 31, the instability criterion of Equation (24) here takes the form *D*_eff_ = 0. We have inserted Equations ((15), (16), (17), (18), (19)) into Equation (32) for *D*_eff_ and searched for the root. The result for the instability line in temperature‐composition space, *T* ^I^(*x*), is

(33) $$T^{I}=4\;T_{\mathrm{plate}}^{C}\;x^{B}\left(1-x^{B}\right)$$

with

(34) $$T_{\mathrm{plate}}^{C}=T_{P}^{C}-\frac{Y\eta^{2}\Omega }{2R}\;\left(\frac{1}{2}+\frac{1+\nu }{1-\nu }\;\;\frac{3\xi^{2}-6\xi +4}{\xi^{3}}\;\right)$$

In the limit ξ → ∞, the instability line coincides with the spinodal for uniaxially constant strain, parameterized by Equation (13), as for the plate with no segregation layer. The limit ξ → 2—just before the layer takes up the entire plate—has the instability at the bulk coherent spinodal, parameterized by Equation 14. For intermediate values of the layer thickness, Figure 7 shows in its part (b) an exemplary map of the instability region in temperature‐composition space (here for the example of ξ = 20), and in its part (c) the variation of $T_{\mathrm{plate}}^{C}$ with the layer thickness.

The transformation mechanism maps of Figure 8 underline that the stiffening by the inert surface layers progressively widens the region of stability of the uniform single‐phase state as the plate thickness is reduced. As argued in Section 6.4, the regions of nucleation and growth and of coherent spinodal decomposition are only affected by the dimensionality of the local mechanical constraint at any point in the bulk of the plate. These regions are therefore consistent with those of Section 6.4.

As a comparison of the graphs of Equations (34) and (30) in Figure 7b reveals, the temperature regime for the buckling instability is more strongly curtailed by the size reduction than the temperature regime for the continuous switchover. This is also apparent in the exemplary transformation mechanism maps of Figure 8. The distinction may be traced back to the way in which the inert outer layers interact with the corresponding strain fields. The plate may remain straight during the continuous switchover, with identical composition strain in bulk and layers. By contrast, bending has the largest strains located in the layers, and this provides the layers enhanced leverage in affecting the bending stiffness. That concept is routinely exploited by mechanical engineers for enhancing the rigidity of I‐beams, and it here leaves a signature in coherent alloy chemistry.

### 7.4 Wetting Transition

Exploring the coherent lithiation of ${\mathrm{FePO}}_{4}$, Cogswell and Bazant^[^ ^35^ ^]^ have pointed out a tendency for strong surface segregation, similar to the present scenario, on certain crystallographic facets. As those authors demonstrated, the instability of the open system to forming the concentrated phase may then take the form of a solid‐state wetting transition. Their theory for a 1D system, of similar geometry as the plate of the present approach, finds the instability at the coherent solubility limit. As the finite‐size particle becomes unstable, they find the chemical potential shifted, relative to that at the bulk coherent solubility limit, by

(35) $$\Delta \mu_{w}=-\frac{1}{\rho_{0}}\sqrt{\kappa_{\mathrm{CH}}\frac{Y\eta^{2}}{1-\nu }\left(x_{0}^{★}-\bar{x}\right)}\;\frac{A}{V}$$

where κ_CH_ is the Cahn–Hilliard gradient energy coefficient^[^ ^86^ ^]^ and $x_{0}^{★}-\bar{x}$ is the difference between the coherent solubility limit and the actual mean solute fraction at the onset of the instability.

Since *A*/*V* = 2/(*t*ξ) for the plate geometry, Equations (27) and (35) agree on a chemical‐potential shift that is inversely proportional to the system dimension. However, the prefactors depend differently on the materials parameters, consistent with the notion that wetting and the surface‐induced stresses represent different interaction mechanisms. The distinction results from different assumption on the interaction terms—whereas the present work ignores gradient energy terms, the theory in [35] assumes a stress‐free boundary condition, neglecting the surface‐induced stress. For Pd‐H inspired materials parameters, a rough estimate (see Section S6, Supporting Information) suggests a stronger response from the present, surface‐induced stress‐based approach compared to that of the wetting mechanism. Gradient energy and surface‐induced stress are both inherent aspects of the scenario at hand.^[^ ^85^, ^86^ ^]^ While an analysis accounting simultaneously for both is beyond the scope of the present work, a future study along that line would be of interest.

## 8 Conclusions

Our analysis of coherent phase stability and coherent transformation mechanisms in interstitial solutions rests *i*) on constrained equilibrium phase diagrams that are informed by geometry‐ and dimensionality‐specific mechanical boundary conditions and *ii*) on elastic instabilities—again geometry‐specific—as implied by sign inversions in open‐system elastic parameters. Somewhat counter to intution, the latter approach derives limits to the stability of a chemically uniform solution from a linear stability analysis in continuum mechanics. We show that this can provide, in a straightforward manner, closed‐form expressions for instability lines in the composition‐temperature domain.

The results of our analysis are displayed in transformation mechanism maps. When capillarity can be neglected, all regions in those maps are bordered by binodal or spinodal lines from constrained‐equilibrium alloy phase diagrams. Closed form expressions are given for the critical temperatures and the spinodals. Our results are consistent among themselves and with several aspects of the state of the art.

We present an example for the impact of capillarity, and show that surface‐induced stresses in the bulk can weaken an effective solute‐solute interaction energy, in proportion to the inverse system size. Here, instability lines have different size‐dependence for canonical versus grand canonical ensembles.

The relevance of geometry is inspected, in our analysis, by comparing infinitely extended systems with beam‐ and plate‐shaped ones, in which lateral stress relaxation at the free surfaces leaves the net stress uniaxial. Other geometries, such as spheroid particles, and rough surfaces can be relevant. Here again, coherency stresses can relax at free surfaces, edges, corners or other asperities, and this will affect the transformation mechanism map. Deformation modes and elastic instabilities for those geometries invite further study.

We demonstrate that coherent transformation mechanism maps differ qualitatively between infinitely extended systems—relevant for numerical simulation using periodic boundary conditions and for theory adopting the Bitter‐Crum theorem—and finite‐size experimental ones. This emphasizes that realistic theory and simulations need to account for the dimensionality.

For finite‐sized systems, exemplified by our beam and plate geometries, we find temperature intervals where the equilibrium phase diagram shows continuous solid solubility, while there are still composition gradients at equilibrium. The gradients emerge from analysis of the open‐system mechanical stability, and they emphasize the complementarity between, on the one hand, the classic, alloy‐chemistry‐based analysis of compositional stability and, on the other hand, a mechanics‐based stability analysis, working with open‐system elastic parameters. Spontaneous buckling due to chemical driving forces presents another aspect of this complementarity.

We confirm an instability of open systems – incoherent or coherent – in the form of a continuous switchover which does not require the formation of a distinct new phase. This instability acts at the chemical spinodals, and it supports the notion of a hysteresis that is “thermodynamic” inasmuch as it reflects the interval of chemical potentials between the spinodals. Yet, there are temperature intervals where incoherent or coherent nucleation and growth can forestall the switchover.

Technological applications of coherent phase‐change systems, for instance for energy storage, stand to benefit from rational design. As a prerequisite therefore, the present study shows how the location of phase transformations in temperature‐composition space and the nature of the transformation can be inferred. The analysis unravels a hierarchy of instabilities, the location and sequence of which depends strongly on the system geometry, the boundary conditions, and the chemical and mechanical materials parameters. In this sense, the present insights, and transformation mechanism maps that can be derived on their basis, may promote advanced energy storage materials with enhanced performance.

## Conflict of Interest

The authors declare no conflict of interest.

## Supporting information

Supporting Information

## Data Availability

The data that support the findings of this study are available from the corresponding author upon reasonable request.

## References

[1] J. D. Eshelby , The Continuum Theory of Lattice Defects, vol. 3, Academic Press, 1956, pp. 79–144.

[2] F. R. d. Boer , W. C. M. Mattens , R. Boom , A. R. Miedema , A. K. Niessen , Cohesion in metals, North‐Holland, Netherlands 1988.

[3] C. Wagner , Acta Metall. 1971, 19, 843.

[4] G. Alefeld , *Hydrogen in metals I ‐ Basic Properties*, volume 28 of *Topics in Applied Physics* , Springer, Berlin 1978.

[5] J. V. G. Alefeld , Hydrogen in metals II ‐ Application Oriented Properties, Topics in Applied Physics, vol. 29, Springer, Berlin 1978.

[6] C. Delacourt , P. Poizot , J.‐M. Tarascon , C. Masquelier , Nat. Mater. 2005, 4, 254.

[7] J. L. Dodd , R. Yazami , B. Fultz , Electrochem. Solid‐State Lett. 2006, 9, A151.

[8] P. Bai , D. A. Cogswell , M. Z. Bazant , Nano Lett. 2011, 11, 4890. 21985573 10.1021/nl202764f

[9] J. W. Cahn , Acta Metall. 1961, 9, 795.

[10] R. Williams , Metall. Trans. A 1980, 11, 247.

[11] Z.‐K. Liu , J. Ågren , Acta Metall. Mater. 1990, 38, 561.

[12] A. L. Roytburd , J. Slutsker , Mater. Sci. Eng., A 1997, 238, 23.

[13] P. W. Voorhees , W. C. Johnson , The Thermodynamics of Elastically Stressed Crystals; in Solid State Physics, (Eds.: H. Ehrenreich , F. Spaepen ), vol. 59, Academic Press, New York 2004, pp. 1–201.

[14] M. Hillert , Phase Equilibria, Phase Diagrams and Phase Transformations: Their Thermodynamic Basis, Cambridge University Press, Cambridge, MA 2007.

[15] J. W. Cahn , R. Kobayashi , Acta Metall. Mater. 1995, 43, 931.

[16] M. Doi , Prog. Mater. Sci. 1996, 40, 79.

[17] D. A. Cogswell , M. Z. Bazant , ACS Nano 2012, 6, 2215. 22304943 10.1021/nn204177u

[18] M. McDowell , S. Lee , W. Nix , Y. Cui , Adv. Mater 2013, 25, 4966. 24038172 10.1002/adma.201301795

[19] L. Chen , F. Fan , L. Hong , J. Chen , Y. Z. Ji , S. L. Zhang , T. Zhu , L. Q. Chen , J. Electrochem. Soc. 2014, 161, F3164.

[20] D. A. Cogswell , M. Z. Bazant , Electrochem. Commun. 2018, 95, 33.

[21] R. B. Schwarz , A. K. Khachaturyan , A. Caro , M. I. Baskes , E. Martinez , J. Mater. Sci. 2020, 55, 4864.

[22] J. M. Rahm , J. Löfgren , P. Erhart , Acta Mater. 2022, 227, 117697.

[23] M. Tang , H.‐Y. Huang , N. Meethong , Y.‐H. Kao , W. C. Carter , Y.‐M. Chiang , Chem. Mat. 2009, 21, 1557.

[24] M. Tang , J. F., Belak , M. R., Dorr , J. Phy. Chem. C. 2011, 115, 4992.

[25] J. W. Cahn , F. Larché , Acta Metall. 1984, 32, 1915.

[26] P. Fratzl , O. Penrose , J. L. Lebowitz , J. Stat. Phys. 1999, 95, 1429.

[27] J. W. Cahn , Philos. Mag. 2013, 93, 3741.

[28] R. B. Schwarz , A. G. Khachaturyan , Phys. Rev. Lett. 1995, 74, 2523. 10057949 10.1103/PhysRevLett.74.2523

[29] X. Xia , A. Afshar , H. Yang , C. M. Portela , D. M. Kochmann , C. V. Di Leo , J. R. Greer , Nature 2019, 573, 205. 31511685 10.1038/s41586-019-1538-z

[30] G. Oyama , Y. Yamada , R.‐i. Natsui , S.‐i. Nishimura , A. Yamada , J. Phys. Chem. C 2012, 116, 7306.

[31] R. Bardhan , L. O. Hedges , C. L. Pint , A. Javey , S. Whitelam , J. J. Urban , Nat. Mater. 2013, 12, 905. 23913172 10.1038/nmat3716

[32] N. J. Weadock , P. W. Voorhees , B. Fultz , Phys. Rev. Mater. 2021, 5, 013604.

[33] C. Delacourt , P. Poizot , S. Levasseur , C. Masquelier , Electrochem. Solid‐State Lett. 2006, 9, A352.

[34] D. Burch , M. Z. Bazant , Nano Lett. 2009, 9, 3795. 19824617 10.1021/nl9019787

[35] D. A. Cogswell , M. Z. Bazant , Nano Lett. 2013, 13, 3036. 23638854 10.1021/nl400497t

[36] R. Malik , F. Zhou , G. Ceder , Nat. Mater. 2011, 10, 587. 21765400 10.1038/nmat3065

[37] H. Liu , F. C. Strobridge , O. J. Borkiewicz , K. M. Wiaderek , K. W. Chapman , P. J. Chupas , C. P. Grey , Science 2014, 344, 1252817. 24970091 10.1126/science.1252817

[38] J. Niu , A. Kushima , X. Qian , L. Qi , K. Xiang , Y.‐M. Chiang , J. Li , Nano Lett. 2014, 14, 4005. 24823479 10.1021/nl501415b

[39] J. Lim , Y. Li , D. H. Alsem , H. So , S. C. Lee , P. Bai , D. A. Cogswell , X. Liu , N. Jin , Y.‐s. Yu , N. J. Salmon , D. A. Shapiro , M. Z. Bazant , T. Tyliszczak , W. C. Chueh , Science 2016, 353, 566. 27493180 10.1126/science.aaf4914

[40] M. Z. Bazant , Faraday Discuss. 2017, 199, 423. 28573280 10.1039/c7fd00037e

[41] N. Nadkarni , E. Rejovitsky , D. Fraggedakis , C. V. Di Leo , R. B. Smith , P. Bai , M. Z. Bazant , Phys. Rev. Mater. 2018, 2, 085406.

[42] B. Koo , J. Chung , J. Kim , D. Fraggedakis , S. Seo , C. Nam , D. Lee , J. Han , S. Jo , H. Zhao , N. Nadkarni , J. Wang , N. Kim , M. Weigand , M. Z. Bazant , J. Lim , Energy Environ. Sci. 2023, 16, 3302.

[43] M. Kamaraj , Sadhana 2003, 28, 115.

[44] A. Lahiri , T. A. Abinandanan , M. P. Gururajan , S. Bhattacharyya , Philos. Mag. Lett. 2014, 94, 702.

[45] F. Wang , K. Yang , M. Ge , J. Wang , J. Wang , X. Xiao , W.‐K. Lee , L. Li , M. Tang , ACS Energy Lett. 2022, 7, 1648.

[46] A. Ulvestad , M. J. Welland , W. Cha , Y. Liu , J. W. Kim , R. Harder , E. Maxey , J. N. Clark , M. J. Highland , H. You , P. Zapol , S. O. Hruszkewycz , G. B. Stephenson , Nat. Mater. 2017, 16, 565. 28092689 10.1038/nmat4842

[47] F. Hayee , T. C. Narayan , N. Nadkarni , A. Baldi , A. L. Koh , M. Z. Bazant , R. Sinclair , J. A. Dionne , Nat. Commun. 2018, 9, 1775. 29720644 10.1038/s41467-018-04021-1 PMC5932065

[48] J. R. Greer , W. D. Nix , Phys. Rev. B 2006, 73, 245410.

[49] J. R. Greer , J. T. M. De Hosson , Prog. Mater. Sci. 2011, 56, 654.

[50] C. Wagner , Z. Phys. Chem. 1943, 193, 386.

[51] J. Weissmüller , C. Lemier , Philos. Mag. Lett. 2000, 80, 411.

[52] N. Meethong , H.‐Y. S. Huang , W. C. Carter , Y.‐M. Chiang , Electrochem. Solid‐State Lett. 2007, 10, A134.

[53] A. Baldi , T. C. Narayan , A. L. Koh , J. A. Dionne , Nat. Mater. 2014, 13, 1143. 25194700 10.1038/nmat4086

[54] S. Syrenova , C. Wadell , F. A. A. Nugroho , T. A. Gschneidtner , Y. A. Diaz Fernandez , G. Nalin , D. Świtlik , F. Westerlund , T. J. Antosiewicz , V. P. Zhdanov , K. Moth‐Poulsen , C. Langhammer , Nat. Mater. 2015, 14, 1236. 26343912 10.1038/nmat4409

[55] R. Griessen , N. Strohfeldt , H. Giessen , Nat. Mater. 2016, 15, 311. 26569476 10.1038/nmat4480

[56] F. Larché , J. W. Cahn , Acta Metall. 1973, 21, 1051.

[57] J. Bonet , R. D. Wood , Nonlinear Continuum Mechanics for Finite Element Analysis, Cambridge University Press, Cambridge; New York, NY 2008.

[58] H. Callen , Thermodynamics and an Introduction to Thermostatistics, Wiley, New York 1985.

[59] The system S of our analysis is a solid solution of charge‐neutral solute atoms. When the fluid reservoir R contains the solute species in the form of ions, then the experimental parameters that afford control over the equilibrium value of μ in S are the activity of the ion in R and the electrode potential.

[60] R. DeHoff , Thermodynamics in Materials Science, 2nd ed., CRC Press, Boca Raton, FL 2006.

[61] Dimensionless variables are defined as follows [69]: Dimensionless temperature $\tau =T/T_{P}^{C}$ with $T_{P}^{C}$ the miscibility gap critical temperature at constant pressure. Dimensionless bending moment m=Md/Iθ with *M* the bending moment, *d* the cantilever thickness in radial direction, *I* the area moment of inertia of the cross‐section, and θ = ρ_0_ω_P_ a characteristic chemical free energy density. Dimensionless curvature *k* = κ*d* with κ the curvature. Dimensionless radial position in a beam ζ =*z*/*d*, with *z* the position coordinate and *z* = 0 at the beam center.

[62] E. Wicke , H. Brodowsky , H. Züchner , in Hydrogen in Metals II, (Eds.: J. Alefeld , G. Völkl ), vol. 29, Springer Berlin Heidelberg, Berlin, Heidelberg 1978, pp. 73–155.

[63] T. B. Flanagan , W. Oates , Annu. Rev. Mater. Sci. 1991, 21, 269.

[64] J. W. Arblaster , Platinum Metals Rev. 2012, 56, 181.

[65] H. Frieske , E. Wicke , Ber. Bunsenges. Phys. Chem. 1973, 77, 48.

[66] T. B. Massalski , H. Okamoto , P. R. Subramanian , L. Kacprzak , Binary alloy phase diagrams, ASM International, Materials Park, OH 1990.

[67] E. Brandes , G. Brook , Smithells Metals Reference Book, Elsevier, Butterworth‐Heinemann, Oxford 2013.

[68] H. Peisl , in Hydrogen in Metals I, (Eds: G. Alefeld , J. Völkl ), vol. 28, Springer, Berlin, Heidelberg 1978, pp. 69–70.

[69] S. Shi , J. Markmann , J. Weissmüller , Phil. Mag. 2017, 97, 1571.

[70] S. Timoschenko , J. Goodier , Theory of Elasticity, 2nd ed., McGraw‐Hill, New York 1951.

[71] J. Weissmüller , S. Shi , Acta Mater. 2022, 227, 117696.

[72] W. C. Johnson , C. S. Chiang , J. Appl. Phys. 1988, 64, 1155.

[73] A. Weidinger , D. Nagengast , C. Rehm , F. Klose , B. Pietzak , Thin Solid Films 1996, 275, 48.

[74] S. Wagner , P. Klose , V. Burlaka , K. Nörthemann , M. Hamm , A. Pundt , ChemPhysChem 2019, 20, 1890. 31106955 10.1002/cphc.201900247

[75] J. Weissmüller , C. Lang , C. Lemier , Scr. Mater. 2001, 44, 1899.

[76] F. Larche , J. W. Cahn , Acta Metall. 1978, 26, 53.

[77] S. Shi , J. Markmann , J. Weissmüller , Proc. Natl. Acad. Sci. U.S.A. 2018, 115, 10914. 30291190 10.1073/pnas.1809355115 PMC6205470

[78] F. C. Larché , J. W. Cahn , Acta Metall. Mater. 1992, 40, 947.

[79] Y. Mishin , J. W. Cahn , Acta Mater. 2016, 117, 197.

[80] M. Gößler , E.‐M. Steyskal , M. Stütz , N. Enzinger , R. Würschum , Beilstein J. Nanotechnol. 2018, 9, 3013. 30591849 10.3762/bjnano.9.280 PMC6296432

[81] C. Mittelstedt , Theory of Plates and Shells, Springer Nature, Berlin 2023.

[82] G. Jerkiewicz , Electrocatalysis 2010, 1, 179.

[83] T. Kuji , Y. Matsumura , H. Uchida , T. Aizawa , J. Alloys Compd. 2002, 330, 718.

[84] C. Lemier , J. Weissmüller , Acta Mater. 2007, 55, 1241.

[85] J. Weissmüller , J. W. Cahn , Acta Mater. 1997, 45, 1899.

[86] J. W. Cahn , J. E. Hilliard , J. Chem. Phys. 1958, 28, 258.

