# Supplementary material for: Coherent Phase Change in Interstitial Solutions: A Hierarchy of Instabilities
Source: Adv Sci (Weinh). 2024 Mar 21;11(21):2308554. doi: 10.1002/advs.202308554 (PMC11151036; doi:10.1002/advs.202308554)
Supplement: Supplementary file 1 — Supporting Information [file ADVS-11-2308554-s001.pdf]

ADVANCED SCIENCE
Open Access

# Supporting Information

for *Adv. Sci.*, DOI 10.1002/advs.202308554

Coherent Phase Change in Interstitial Solutions: A Hierarchy of Instabilities

*Jörg Weissmüller**

# SUPPLEMENTARY INFORMATION FOR "COHERENT PHASE CHANGE IN INTERSTITIAL SOLUTIONS – A HIERARCHY OF INSTABILITIES"

By Jörg Weissmüller

*Hamburg University of Technology and Helmholtz-Zentrum Hereon*

## S1. EXPLICIT DISPLAY OF THE EQUATIONS OF STATE FOR THE CHEMICAL POTENTIAL AT CONTROLLED STRAIN

The equations of state for the chemical potential of the regular solution at controlled strain are obtained by substituting Eqs 8 or 9 for $P$ in the equation of state for controlled pressure, Eq 5, and by concatenating the linear $x$-dependent terms into a new, effective $\omega$, as indicated by Eq 10. For uniaxially or biaxially controlled strain, the results are

$$\begin{aligned}\tilde{\mu}_{\rm ua}(T,x,\varepsilon) &= \omega_{\rm P}(1-2x) + RT\ln\frac{x}{1-x} \\ &\quad -\eta\Omega Y(\varepsilon-\eta x) \qquad \text{(S1)}\\ &= \omega_{\rm ua}(1-2x) + RT\ln\frac{x}{1-x} - \eta\Omega Y(\varepsilon - \frac{1}{2}\eta)\end{aligned}$$

and

$$\begin{aligned}\tilde{\mu}_{\rm ba}(T,x,\varepsilon) &= \omega_{\rm P}(1-2x) + RT\ln\frac{x}{1-x} \\ &\quad -2\eta\Omega\frac{Y}{1-\nu}(\varepsilon-\eta x) \qquad \text{(S2)}\\ &= \omega_{\rm ba}(1-2x) + RT\ln\frac{x}{1-x} - \frac{2\eta\Omega Y}{1-\nu}(\varepsilon - \frac{1}{2}\eta)\end{aligned}$$

respectively. The rescaled solute-solute interaction energy parameters, $\omega_{\rm ua}$ and $\omega_{\rm ba}$, are given by Eqs 11 and 12, respectively.

Fixing the strain $\varepsilon$ is seen to result in $(i)$ the rescaling of the solute-solute interaction energy $\omega$ and $(ii)$ a shift of $\mu$ (for any given $x$) by a constant that depends on $\varepsilon$ and on the dimensionality of the constraint. Otherwise, the variation of $\mu$ with $x$ remains formally that of a regular solution. In each case, the constrained equilibrium phase diagram then agrees with that of a conventional regular solution. The critical temperature of that phase diagram's miscibility gap is reduced (compared to the constant-pressure scenario) in proportion to $\omega$ and, thereby, by an amount that depends on the dimensionality of the constraint.

## S2. CONSISTENCY CHECK: IMPLICATIONS OF THE BITTER-CRUM THEOREM FOR THE FREE ENERGY IN CLOSED AND IN OPEN SYSTEMS

The total free energy $\mathcal{G}^{\sf S}$ of the solid body $\sf S$ (the system, or sample) can be represented by a volume integral over a local Gibbs free energy density, $\Psi$ (free energy per referential volume), see Eq 1. With attention to systems in the absence of external load (superscript 0), we assume that the net free energy density, $\Psi = \Psi^0$, can be decomposed into two additive terms, the "chemical" (combined electronic and entropic contributions) free energy in $\Psi_{\rm chm}$ and the elastic (misfit and coherency) strain energy in $\Psi_{\rm mft}$,

$$\Psi^0(T,\tilde{x}(\mathbf{r}),\mathbf{r}) = \Psi_{\rm chm}(T,x(\mathbf{r})) + \Psi_{\rm mft}(T,\tilde{x}(\mathbf{r}),\mathbf{r})\,. \qquad \text{(S3)}$$

Here $\tilde{x}(\mathbf{r})$ is the position- ($\mathbf{r}$-) dependent composition field in $\sf S$, which reduces to a constant, $\tilde{x}(\mathbf{r}) = \bar{x}$, in the special case of a uniform solution. The energy density of coherency stresses is formally and implicitly accounted for by making $\Psi^0$ a functional of the composition field.

The Bitter-Crum theorem [25–27] states that the misfit strain energy due to centres of dilatation – for instance, misfitting solute atoms or precipitates – in a uniform, isotropic and linear elastic solid with no free surfaces is independent of the spatial arrangement of the centres. This may be expressed as

$$\mathcal{G} = \int_{\sf S} \Psi_{\rm chm}(T,x(\mathbf{r}))\mathrm{d}V + \Psi_{\rm mft}(T,\bar{x})V^{\sf S}\,, \qquad \text{(S4)}$$

with $\bar{x}$ the mean solute fraction, namely $\bar{x} = N/N_0$ where $N$ and $N_0$ denote the total amounts of solute and of interstitial sites, respectively, in $\sf S$. $V^{\sf S}$ denotes the volume of $\sf S$.

Equation S4 implies specifically that the misfit strain energy – as embodied in $\Psi_{\rm mft}(T,\bar{x})$ – cannot contribute to a possible driving force for the decomposition of a closed (constant $\bar{x}$) coherent system. Instead, the miscibility gap in the closed-system alloy phase diagram here derives from the chemical and entropic free energy density, $\Psi_{\rm chm}(T,x)$, alone. For the present choice of model and materials parameters, that phase diagram is the biaxially-controlled-strain phase diagram of Fig 1. This follows, since our biaxially-controlled-strain scenarios is Bitter-Crum compatible. Comparison with the conventional, constant-pressure phase diagram in the figure illustrates that the equilibrium alloy phase diagram may be expected drastically modified in Bitter-Crum-type systems, compared to incoherent ones.

Open systems present a different case. Here, exchange of solute with an external reservoir does change $\bar{x}$. Specifically, if the system where to remain uniform – as is possible, in principle, during continuous switchover – then Eq S4 would become

$$\begin{aligned}\mathcal{G} &= \int_{\sf S} \Psi_{\rm chm}(T,\bar{x})\mathrm{d}V + \Psi_{\rm mft}(T,\bar{x})V^{\sf S} \qquad \text{(S5)}\\ &= \Psi^0(T,\bar{x})\,V^{\sf S}\,.\end{aligned}$$

In other words, the conditions for the continuous switchover instability in the open, Bitter-Crum-type coherent system derive from the net free-energy function $\Psi^0(T,x)$; this is the identical condition as in the incoherent system. By contrast, the constrained equilibrium phase diagram of the closed, Bitter-Crum-type coherent system derives from $\Psi_{\mathrm{chm}}(T,x)$ alone, and this leads to substantially different stability regions.

For open systems, any energy barrier to transformations that arises from the misfit or coherency strain energy $\Psi_{\mathrm{mft}}(T,\bar{x})$ scales with the sample volume, see the second term on the right-hand-side of Eq S4. As was pointed out in [28], such barriers are then typically macroscopic and cannot be overcome by thermal activation.

## S3. CONSISTENCY CHECK: SCHWARZ-KHACHATURYAN TYPE INSTABILITY IN AN EULER-BERNOULLI BEAM

### A. Mechanics of a solid-solution Euler-Bernoulli beam

The approach in this section is based on [71]. We consider the mechanics of a solid-solution beam. The beam is free of external load, but a trend for chemical disproportionation embodied in the the free-energy function of the solution may set up radial composition gradients that result in deformation. The thickness in radial direction and the width are $d$ and $w$, respectively, the deformation mode under consideration is bending though a circular arc of curvature $\kappa$, and within the Euler-Bernoulli approximation the stress is uniaxial along the long axis of the beam. With the origin of the radial coordinate $z$ in the centre of the beam, the strain, $\varepsilon$, specified relative to the stress-free and pure (no solute) matrix, is

$$\varepsilon = (z - z_0)\kappa\,, \tag{S6}$$

where the neutral fibre position is related to the mean solute fraction $\overline{x}$ by [71]

$$z_0 = \overline{x}\eta/\kappa\,. \tag{S7}$$

The axial stress, $\sigma$, relates to the strain by

$$\sigma = Y(\epsilon - \eta x)\,, \tag{S8}$$

and the bending moment is

$$M = w\int_{-d/2}^{+d/2} z\sigma \mathrm{d}z\,. \tag{S9}$$

We now evaluate the strain energy of a beam containing two phases of uniform compositions $x_1$ and $x_2$ at solid fractions $1-\varphi$ and $\varphi$, respectively. Solving Eq S9 for $M=0$ while accounting for Eqs S7 and S8, we obtain the curvature at equilibrium as

$$\kappa = \frac{6}{d}(x_2 - x_1)\eta\varphi(1-\varphi)\,. \tag{S10}$$

This result provides the strain- and stress fields via Eqs S6 and S8. The curvature varies parabolically with the phase fraction, with maximum curvature at $\varphi = 0.5$.

Integrating the mechanical energy density, $^1/_2\,\sigma^2/Y$, over the beam volume, we obtain the net mechanical (coherency strain) energy density (per Lagrangian volume) as

$$\Psi_{\mathrm{coh}} = \frac{Y}{2}\left(x_2 - x_1\right)^2\eta^2\varphi(1-\varphi)(1-3\varphi(1-\varphi))\,. \tag{S11}$$

The coherency strain energy emerges as fourth-order polynomial in the phase fraction; this is expected in view of the parabolic variation of the curvature with $\varphi$, Eq S10, combined with quadratic dependency of the local strain energy density on the local strains (which scale with $\kappa$).

### B. Coherent instable formation of the concentrated second phase

Consider the uniform open system $\mathsf{S}$ at equilibrium with a reservoir $\mathsf{R}$ at the external chemical potential $\mu^{\mathsf{R}}$. As is noted in Sec VI A of the main text, the composite system $\mathsf{C}$ – namely $\mathsf{S}$ plus $\mathsf{R}$ – is at fixed amount of solute and so, at equilibrium, $\mathsf{C}$ minimizes its Gibbs free energy. Here, contrary to the uniform $\mathsf{S}$ of Sec VI A, we allow for $\mathsf{S}$ to be dual phase, namely containing a precipitate.

When an amount $\delta N$ of solute is transferred from $\mathsf{R}$ to $\mathsf{S}$, the Gibbs free energy change of $\mathsf{C}$ is

$$\delta\mathcal{G}^{\mathsf{C}} = \left(\frac{\partial\tilde{\mathcal{G}}^{\mathsf{S}}}{\partial N} - \mu^{\mathsf{R}}\right)\delta N \tag{S12}$$

Here, $\tilde{\mathcal{G}}^{\mathsf{S}}(T,N_0,N)$ is the equation of state for $\mathcal{G}$ in $\mathsf{S}$; $N_0$ denotes the amount of interstitial sites, which is here fixed. Equilibrium requires $\delta\mathcal{G}^{\mathsf{C}} = 0$ during the process under consideration. This implies $\mu = \partial\tilde{\mathcal{G}}^{\mathsf{S}}/\partial N = \mu^{\mathsf{R}}$ for the chemical potential in the initially uniform system.

The net free energy, $\mathcal{G}^{\mathsf{S}}_{\mathrm{tp}}$, of the two-phase coherent system containing the matrix at solute fraction $x_1$ and a phase fraction $\varphi$ of the new phase of solute fraction $x_2$ is

$$\begin{aligned}\mathcal{G}^{\mathsf{S}}_{\mathrm{tp}} = N_0\Omega\left[(1-\varphi)\tilde{\Psi}^0(x_1) + \varphi\tilde{\Psi}^0(x_2)\right]\\ +N_0\Omega\Psi_{\mathrm{coh}}(x_1,x_2,\varphi)\,.\end{aligned} \tag{S13}$$

with $\tilde{\Psi}^0(x)$ the equation of state for the stress-free free energy.

At equilibrium with the reservoir, and right before the onset of the instability, the system is uniform at composition $x_1$ and chemical potential $\mu_1 = \mu^{\mathsf{R}}$. For the precipitation of the second phase of solute fraction $x_2$ to

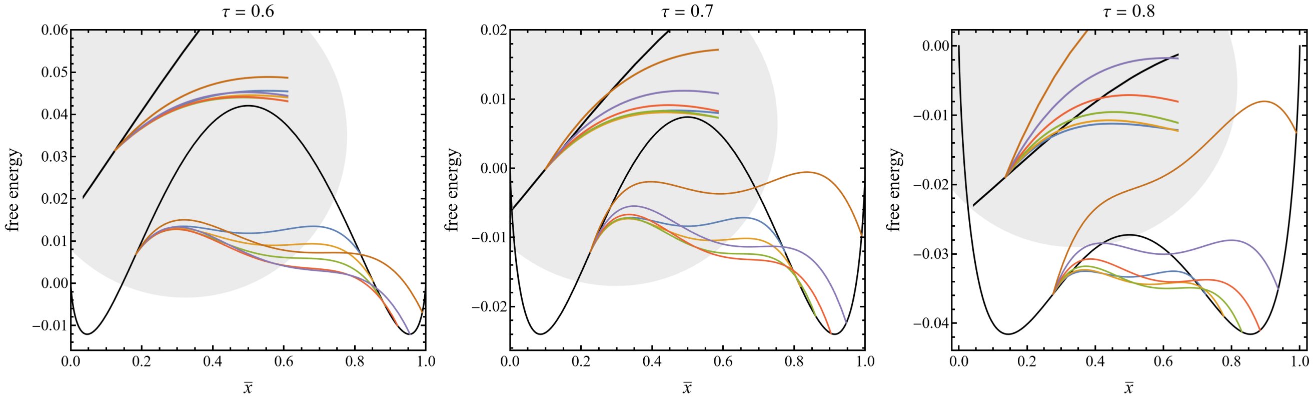


FIG. S1. Analyzing criteria for coherent instable formation of a concentrated second phase in a more dilute matrix, here for precipitation on one side of a curved beam. Graphs of free energy versus mean solute fraction $\bar{x}$ at (from left to right) temperatures $\tau = 0.6$, 0.7 and 0.8. Black: total free energy of the uniform solution. Colored: free energies of coherent two-phase states, Eq S13 with Eq S11 for the coherency strain energy. For the example, the matrix is at the dilute-phase spinodal of the respective temperature. The precipitates are at various solid fractions, as evidenced by the endpoints of the lines. Inserts underlined in gray: enlarged views of detail next to the spinodal compositions, representative of the onset stage of precipitation. System is unstable with respect to precipitation if at least one of the two-phase free energy graphs starts out below the total free energy graph. In the figure, this happens for $\tau$ =0.6 and 0.7, but not 0.8. Results for a long thin beam and formation of a concentrated layer on one side. Materials parameters as in Sec III.

be energetically favorable, it is required that $\delta\mathcal{G}^{\mathsf{C}} < 0$. Thus, as was shown by Schwarz and Khachaturyan [28],

$$\frac{1}{N_0}\frac{\mathrm{d}\mathcal{G}^{\mathsf{S}}_{\mathrm{tp}}}{\mathrm{d}\bar{x}}\bigg|_{x_1,x_2,\varphi\to 0} \leq \mu^{\mathsf{R}}\,. \tag{S14}$$

Here, the derivative is considered in the limit $\varphi \to 0$, and

$$\bar{x} = (1-\varphi)x_1 + \varphi x_2\,. \tag{S15}$$

In view of Eq S13, the derivative on the left-hand-side of Eq S14 is

$$\frac{1}{N_0}\frac{\mathrm{d}\mathcal{G}^{\mathsf{T}}_{\mathrm{tp}}}{\mathrm{d}\bar{x}}\bigg|_{x_1,x_2,\varphi\to 0} = \frac{\Omega}{x_2-x_1}\left(\Psi^0(x_2) - \Psi^0(x_1) + \frac{\mathrm{d}\Psi_{\mathrm{coh}}}{\mathrm{d}\varphi}\bigg|_{x_1,x_2}\right)\,. \tag{S16}$$

Instability against formation of the second phase prevails whenever there are combinations of $x_1$, $x_2$ (with $\mu(x_1) = \mu^{\mathsf{R}}$) that satisfy the inequality of Eq S14.

We have numerically evaluated the criterion of Eqs S14 - S16 using the coherency strain energy of Eq S11 along with the regular solution model and the materials parameters of Sec III. For each out of a set of temperatures, the algorithm searched for the smallest $x_1$ for which the equations can be satisfied for any $x_2 > x_1$. That $x_1$ value marks the onset of matrix instability to precipitation as $\mu^{\mathsf{R}}$ is increased at the selected $T$. Figure S1 illustrates the free energy functions underlying the instability criterion, and how the criterion was exploited for identifying conditions of instability.

The red-shaded regions in Fig S2 show regimes, in the space of matrix composition and temperature, where the inequality can be satisfied for precipitates with any composition. Also shown is the constant-axial-strain binodal. The two graphs agree precisely.

Numerically, it is found that matrices outside of the chemical spinodal (dashed line in Fig S2) can be unstable against coherent precipitation when $\tau < 0.721$. Coherent precipitation is then the first instability that is met when $\mu$ of the open system is increased starting from the dilute phase, see the transformation mechanism maps of Fig 4(c). At higher temperatures, the spinodal is encountered first and the system will transform by continuous switchover.

## S4. PROCEDURES: SPONTANEOUS BUCKLING OF A BEAM AT CONSTANT NET SOLID FRACTION

We used the numerical procedures of [71, section 8.3.2] for deriving bending moment-curvature relations of beams at constant mean solute fraction $\bar{x}$ and at uniform (though not necessarily constant) chemical potential $\mu$. An array of typically 100 nodes, equidistant in radial direction, represented the radial composition- and strain field in the beam. At any value of the dimensionless curvature $k$, the neutral fibre position and, thereby, the strain profile are known a priori from Eq S7. However, the value of the chemical potential varies as the function of the curvature, and determining its value requires numerical evaluation.

The analysis proceeded as follows [71]: *i*) determine the composition field at equilibrium subject to the local strain and to the current value of the chemical potential, using Eq S1, *ii*) integrate to obtain the net composition,

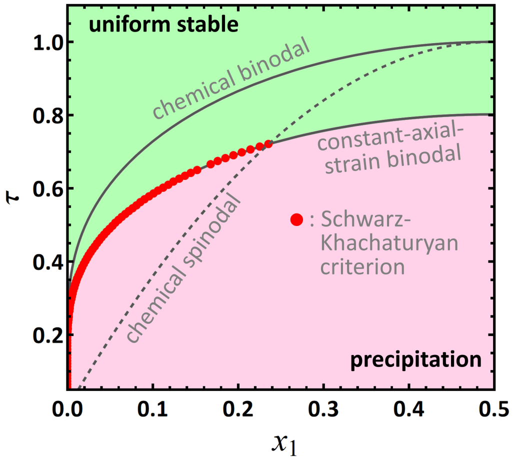


FIG. S2. Coherent precipitation by forming a thin layer of the new phase at one side of a thin, beam-shaped sample that can bend in response; open-system case. Green, regions in the space of temperature ($\tau$) and matrix solute fraction ($x_1$) where the matrix is stable against the coherent precipitation of a second phase of any composition $x_2 > x_1$. Red, regions of instability. Red circles: onset of Schwarz-Khachaturyan-type instability. Note the agreement with the constant-axial-strain binodal. Also shown are the chemical binodal and chemical spinodal. Note that continuous switchover will act beyond the chemical spinodal, preventing uniform stable states and obliterating the need for coherent precipitation; this is not shown in the figure. Results for bending instability in a long, thin beam. Materials parameters motivated by Pd-H, see Sec III.

*iii*) correct the chemical potential value according to the deviation between actual and nominal net composition, *iv*) repeat this procedure until actual and nominal net composition agree relatively to better than $10^{-3}$ as the convergence criterion. The underlying assumptions and equations of state are those of our Sec III and Sec **S3** A.

Combinations of stress, temperature, and chemical potential in which two separate phases can exist require attention. We started the computation at one extreme of the curvature range under investigation, where the spatial distribution of the stable phases was uniquely defined in each case. At any given position, as the curvature evolved, the phase from the prior curvature step was maintained as long as a solution for that phase existed (i.e., until encountering a spinodal); only then was the system allowed to transform to the other phase at that position. This emulates thermodynamic hysteresis. The complete set of results, covering the interval from negative to positive maximum curvature, was then inspected for symmetry as a prerequisite for physically meaningful results.

## S5. STRESS BALANCE AND WORK OF DEFORMATION IN A PLATE WITH INERT SURFACE LAYERS

The plate is taken to consist of the bulk $\mathsf{B}$ and the layer $\mathsf{L}$, and the compositions are considered at fixed values $x^{\mathsf{B}}$ and $x^{\mathsf{L}}$. This leads to stress-free strains $\eta x^{\mathsf{B}}$ and $\eta x^{\mathsf{L}}$. Coherency enforces the in-plane strain, $\varepsilon_{\|}$, to be uniform. As the composition is considered fixed, the in-plane stress in each region can be taken as

$$\sigma_{\|} = B(\varepsilon_{\|} - \eta x) \tag{S17}$$

with $B$ the conventional, constant-composition biaxial modulus and $x$ the local solute fraction. The volumetric mean in-plane stress of the plate is then

$$\begin{aligned}\langle\sigma_{\|}\rangle_{\mathrm{V}} &= \frac{1}{d}\left((d-2t)\sigma_{\|}^{\mathsf{B}} + 2t\sigma_{\|}^{\mathsf{L}}\right)\\ &= \frac{B}{d}\left((d-2t)(\varepsilon_{\|} - \eta x^{\mathsf{B}}) + 2t(\varepsilon_{\|} - \eta x^{\mathsf{L}})\right)\end{aligned} \tag{S18}$$

As there is no external load, mechanical equilibrium requires that $\langle\sigma_{\|}\rangle_{\mathrm{V}} = 0$. Inserting this into Eq S18 and solving for $\varepsilon_{\|}$, one finds the in-plane strain at elastic equilibrium as

$$\varepsilon_{\|} = \eta(x^{\mathsf{B}} + \frac{2}{\xi}(x^{\mathsf{L}} - x^{\mathsf{B}})) \tag{S19}$$

with $\xi = d/t$. Equation S17 then immediately supplies the in-plane stress in the bulk, Eq 26 in the main text.

For the linear stability analysis, the work of deformation in the limit of small curvature is obtained in Eq 31 of the main text. A more detailed version of this equation shows how the different elastic parameters in layers and bulk enter the derivation:

$$\begin{aligned}W &= A\int_{-d/2}^{d/2}\Psi_{\mathrm{mech}}\mathrm{d}z = A\int_{-d/2}^{d/2}\frac{1}{2}\sigma\varepsilon\mathrm{d}z\\ &= 2A\left(\int_{0}^{d/2-t}\frac{1}{2}\overset{*}{D}\varepsilon^2\mathrm{d}z + \int_{d/2-t}^{d/2}\frac{1}{2}D\varepsilon^2\mathrm{d}z\right)\\ &= A\left(\int_{0}^{d/2-t}\overset{*}{D}\kappa^2z^2\mathrm{d}z + \int_{d/2-t}^{d/2}D\kappa^2z^2\mathrm{d}z\right)\\ &= \frac{A\kappa^2}{24}\left(\overset{*}{D}d^3 - 2\left(\overset{*}{D} - D\right)\left(2d^2t - 6dt^2 + 4t^3\right)\right),\end{aligned} \tag{S20}$$

where $\varepsilon = \kappa z$ was used for the bending strain. Substituting $d = \xi t$ provides the leastic parameter in the form of 32 of the main text.

## S6. WETTING INSTABILITY OF REFERENCE [35]–ESTIMATING THE CHEMICAL POTENTIAL SHIFT

Here, we estimate the magnitude of the size -dependent shift in chemical potential for the solid-state wetting scenario of reference [35], for comparison with the chemical-potential shift of the present work. Consistent with the

exemplary scenario underlying the figures of the present paper, we base the comparison on the example of the the Pd-H-informed regular solution.

Equation 35 in the main text indicates the shift in chemical potential at instability, as predicted by the theory in [35] (equations (2) and (21) of that work with equation (12) of [9] for the strain energy, as suggested in [35]).

Estimating the magnitude of the pre-factor in front of the $A/V$-term, which embodies the size dependence, requires a numerical value for the gradient energy coefficient $\kappa_{\rm CH}$. This is obtained from $\kappa_{\rm CH} = \omega\lambda^2/2\rho_0$ and, for the regular solution, $\lambda = r_{\rm NN}/\sqrt{3}$ with $r_{\rm NN} = a_0/\sqrt{2}$ the atomic nearest-neighbor spacing [86]. With the Pd-H materials parameters in Sec III, the result is $\kappa_{\rm CH} \approx 1.3 \times 10^{-11}$J/m.

The factor $(x_0^\star - \overline{x})$ in Eq 35 represents the difference between the concentration at the coherent solubility limit (coherent binodal) and the mean solid fraction in the system. As the wetting layer will be concentrated, whereas the bulk of the sample remains more dilute, we may estimate that term as in the order of 1/2. Using again the Pd-H-informed materials parameters, we may then approximate Eq 35 for the size-dependent shift in chemical potential due to a wetting transition as

$$\Delta\mu_{\rm w} \approx 6 \times 10^{-7} \frac{\rm Jm}{\rm mol} \frac{A}{V} \,. \qquad \text{(S21)}$$

Let us now compare the just-mentioned estimate to the shift due to the tangential coherency stresses, as analyzed in the present work. For the plate geometry, which is common to [35] and the present work, we have $A/V = 2/(\xi t)$. Thus, Eq 27 can be represented as

$$\Delta\mu^{\sf B} = 3\eta\Omega P = 2t\frac{Y\eta^2\Omega}{1-\nu} \left(x^{\sf B} - x^{\sf L}\right) \frac{A}{V} \,. \qquad \text{(S22)}$$

Using again the Pd-H informed materials parameters, along with $t = 1.0$nm [55], and setting $x^{\sf L} = 1$ and $x^{\sf B} = 1/2$ for two-phase coexistence near $T^{\rm C}$, we obtain

$$\Delta\mu^{\sf B} \approx 5 \times 10^{-6} \frac{\rm Jm}{\rm mol} \frac{A}{V} \,. \qquad \text{(S23)}$$

A comparison of Eqs S21 and S23 suggests for the present example, specific to a metal hydride, that the action of the tangential stresses from the segregation layer may be noticeably stronger than the action of the solid-state wetting transition.

## BIBLIOGRAPHY

References cited here in the Supporting Information are shown in the bibliography of the main document.
